# *In Vivo* Pravastatin Treatment Reverses Hypercholesterolemia Induced Mitochondria-Associated Membranes Contact Sites, Foam Cell Formation, and Phagocytosis in Macrophages

**DOI:** 10.3389/fmolb.2022.839428

**Published:** 2022-03-15

**Authors:** Leandro Henrique de Paula Assis, Gabriel de Gabriel Dorighello, Thiago Rentz, Jane Cristina de Souza, Aníbal Eugênio Vercesi, Helena Coutinho Franco de Oliveira

**Affiliations:** ^1^ Department of Structural and Functional Biology, Institute of Biology, State University of Campinas, Campinas, Brazil; ^2^ Department of Clinical Pathology, Faculty of Medical Sciences, State University of Campinas, Campinas, Brazil

**Keywords:** hypercholesterolemia, statin, macrophage, mitochondria-associated membrane, foam cell, phagocytosis

## Abstract

Statins are successful drugs used to treat hypercholesterolemia, a primary cause of atherosclerosis. In this work, we investigated how hypercholesterolemia and pravastatin treatment impact macrophage and mitochondria functions, the key cell involved in atherogenesis. By comparing bone marrow-derived macrophages (BMDM) of wild-type (WT) and LDL receptor knockout (LDLr^−/−^) mice, we observed hypercholesterolemia increased the number of contact sites at mitochondria-associated endoplasmic reticulum (ER) membranes (MAMs), enhanced mitochondrial hydrogen peroxide release, altered the gene expression of inflammatory markers, and increased oxidized LDL (ox-LDL) uptake and phagocytic activity. Three months of *in vivo* pravastatin treatment of LDLr^−/−^ mice reversed the number of contact sites at the MAM, ox-LDL uptake, and phagocytosis in LDLr^−/−^ BMDM. Additionally, pravastatin increased BMDM mitochondrial network branching. In peritoneal macrophages (PMs), hypercholesterolemia did not change MAM stability, but stimulated hydrogen peroxide production and modulated gene expression of pro- and anti-inflammatory markers. It also increased mitochondrial branching degree and had no effects on ox-LDL uptake and phagocytosis in PM. Pravastatin treatment increased superoxide anion production and changed inflammation-related gene expression in LDLr^−/−^ PM. In addition, pravastatin increased markedly the expression of the mitochondrial dynamics-related genes Mfn2 and Fis1 in both macrophages. In summary, our results show that hypercholesterolemia and pravastatin treatment affect macrophage mitochondria network structure as well as their interaction with the endoplasmic reticulum (ER). These effects impact on macrophage conversion rates to foam cell and macrophage phagocytic capacity. These findings associate MAM stability changes with known mechanisms involved in atherosclerosis progression and resolution.

## Introduction

Atherosclerosis is a silent and chronic inflammatory illness characterized by cholesterol-enriched plaque deposition in the arteries (Goldstein and Brown, 2015). Atherosclerosis is the leading cause of cardiovascular diseases, including acute myocardial infarction and stroke that together account for a quarter of deaths worldwide (World Health Organization, 2020). Elevated low-density lipoprotein (LDL) levels in the plasma (hypercholesterolemia) trigger atherogenesis. This condition favors LDL retention in the subendothelial space of the arteries, followed by lipoprotein oxidation. Oxidized LDL (ox-LDL) activates the endothelium, which secretes chemokines and activates an inflammatory response. Immune cells, mainly monocytes, infiltrate into the arterial intima, differentiate into macrophages that take up ox-LDL, and become cholesterol-laden foam cells (Brown and Goldstein, 1983), the hallmark of atherosclerosis (Steinberg and Witztum, 2010; Gao et al., 2017). These activated macrophages secrete pro-inflammatory cytokines (Chinetti-Gbaguidi et al., 2015), perpetuating the inflammation.

The discovery of inhibitors of cholesterol synthesis in the ‘70s and the later commercial launch of statins (Endo et al., 1976) led to a marked drop in morbidity and mortality associated with cardiovascular diseases (Hong and Lee, 2015; Chou et al., 2016). Statins reduce plasma cholesterol levels and atherosclerotic plaque vulnerability (Nilsson, 2017), as well as tissue and systemic inflammation (Antonopoulos et al., 2012). However, statins’ side effects may affect muscle and other tissues (Ward et al., 2019). These undesirable effects depend on statin class and dose, with the hydrophobic drugs being more toxic than the hydrophilic ones (Velho et al., 2006; Kobayashi et al., 2008). Mechanistic studies on statin toxicity suggest mitochondria as a primary target. Statins use may cause mitochondrial respiration inhibition, increased oxidant generation, and mitochondrial permeability transition in the muscle (Mullen et al., 2011; Kwak et al., 2012; la Guardia et al., 2013; Schirris et al., 2015; Busanello et al., 2017), liver (Velho et al., 2006; Mullen et al., 2011; Marques et al., 2018), brain (Fišar et al., 2016), platelet (Vevera et al., 2016), and endothelium (Broniarek and Jarmuszkiewicz, 2018). *In vitro*, statins decrease cell line viability (Oliveira et al., 2008; Costa et al., 2013; Lorza-Gil et al., 2019), including in macrophage models (Croons et al., 2010), in a dose-dependent manner.

The endoplasmic reticulum (ER) membrane fluidity is influenced by its free cholesterol content, which changes as macrophages become foam cells during atherogenesis. This phenomenon causes ER stress, depletes ER calcium stores, and can lead to apoptosis (Feng et al., 2003; DeVries-Seimon et al., 2005; Zhang and Kaufman, 2008). ER membrane fluidity influences protein-protein interactions involved in inter-organelle contact sites (Phillips and Voeltz, 2016), especially if they localize in cholesterol-enriched microdomains (Garofalo et al., 2016). Mitochondria-associated ER membranes (MAMs) are conserved in eukaryotes and participate in many intracellular processes (Lopez-Crisosto et al., 2017; Csordás et al., 2018; Rieusset, 2018). This includes the synthesis and transfer of triacylglycerols and phospholipids (Rusiñol et al., 1994; Shiao et al., 1995; Yeo et al., 2021), mitochondria dynamics (Friedman et al., 2011; Guo et al., 2018), reactive oxygen species signaling (Booth et al., 2021), and calcium flux between organelles and mitochondrial bioenergetics functions (Gomez-Suaga et al., 2017; Filadi et al., 2018). Previous reports have shown altered MAM stability in conditions of obesity (Arruda et al., 2014), diabetes (Tubbs et al., 2014; Thivolet et al., 2017), rheumatoid arthritis, coronary artery disease (Zeisbrich et al., 2018), and amyotrophic lateral sclerosis (Sakai et al., 2021).

We hypothesized that hypercholesterolemia and chronic statin treatment might affect macrophage function, particularly regarding mitochondria structure, function, and interaction with the ER. Thus, we evaluated MAM stability, mitochondrial network morphology, bioenergetics, redox state, oxidant production, macrophage inflammatory state, and function. We used two macrophage sources, bone marrow-derived macrophages (BMDMs) and thioglycolate-elicited peritoneal macrophages (PMs).

## Materials and Methods

### Animals and *In vivo* Treatment

C57BL/6J wild-type (WT) (Jackson Laboratory, 664, Bar Harbor, ME) and LDL receptor knockout (LDLr^−/−^) male mice (B6.129S7-Ldlr < tm1Her./J, homozygous for Ldlr < tm1Her) (Jackson Laboratory, 2,207) were provided by the Multidisciplinary Center for Biological Research in Laboratory Animals (CEMIB) at the State University of Campinas, Campinas, SP, Brazil. Animals were maintained under controlled conditions (22 ± 2°C and a 12:12 h light-dark cycle) and with free access to standard AIN/93M diet (Prag-Soluções, Jaú, Brazil) and filtered water. Pravastatin studies consisted of treating thirty-day-old male mice with 400 mg/l pravastatin sodium (Sanofi Medley, Campinas, Brazil) diluted in drinking water for 3 months (Lorza-Gil et al., 2016). The estimated pravastatin dose of 40 mg/kg body weight/day was based on the average drinking water consumption rate of 3.5 ml/day. Control mice received filtered water without pravastatin. At the end of treatment, four-month-old male mice were euthanized by decapitation under isoflurane (Isoforine, Cristália Produtos Químicos Farmacêuticos Ltda., Lindóia, Brazil) anesthesia.

### L929 Conditioned Medium

Murine L929 cells (ATCC, CCL-1, Manassas, VA) were maintained in Dulbecco’s Modified Eagle Medium High Glucose Plus (DMEM, LGC Biotecnologia, BR-30356-05, Cotia, Brazil) containing 1 mM sodium pyruvate and 25 mM glucose and supplemented with 10% (*v/v*) heat-inactivated Horse Serum, New Zealand Origin (Gibco, 16050122, Waltham, MA) at 37°C and 5% (v/v) CO_2_ according to the manufacturer’s instructions. L929 cells were seeded in a 150 mm diameter Tissue Culture Dish (Falcon, 353025, Corning, NY) and allowed to grow until 80% area confluence. Then, the medium was completely replaced and the cells were incubated with 50 ml of DMEM (Vitrocell, D0069, Campinas, Brazil) containing 25 mM glucose, 1 mM sodium pyruvate, 4 mM L-glutamine, and 2% (*v/v*) heat-inactivated fetal bovine serum (Vitrocell, 11) for 3 days at 37°C and 5% (*v/v*) CO_2_. This L929 conditioned medium was collected, sterilized using a 0.22 µm filter, and frozen at −20°C before use.

### Bone Marrow-Derived Macrophages

Bone marrow from LDLr^−/−^ male mouse femurs was flushed using an L929 conditioned medium as mentioned above. The cellular suspension was centrifuged at 1,000 × g at room temperature, and the supernatant was discarded. Pelleted cells were incubated with 1.0 ml Red Blood Cell Lysing Buffer Hybri-Max (Sigma-Aldrich, R7757, St. Louis, MO) for 3 min at room temperature to lyse red blood cells. Lyses were stopped using 1.0 ml of Dulbecco’s Phosphate Buffered Saline solution (Sigma-Aldrich, D8537). Cells were centrifuged at 1,000 × g at room temperature, and the supernatant was discarded. Then, the bone marrow cells were resuspended in a differentiation medium: 69% (v/v) DMEM, 20% (v/v) L929 conditioned medium, 1% (v/v) Penicillin–Streptomycin Mixture (Lonza, 17-602F, Basel, CH), and 10% (v/v) fetal bovine serum (Vitrocell, 11). Cells were plated in a 100 mm diameter Petri Dish (Nest Biotechnology, 753001, Wuxi, China) and incubated at 37°C and 5% (v/v) CO_2_ for 7 days for full differentiation, changing half part of the differentiation medium at day 3. Bone marrow-derived macrophages (BMDMs) were carefully detached using 1.5 ml Accutase Cell Detachment solution (Sigma-Aldrich, SCR005), counted in a hemocytometer, and plated in a 24-well plate (Nest Biotechnology, 702001) or 96-well plate (Greiner Bio-one, 655090, Kremsmünster, AT) for further analysis.

### Peritoneal Macrophage Isolation

1 ml of 3% (*m/v*) sodium thioglycolate solution was injected intraperitoneally in C57BL/6J and LDLr^−/−^ male mice 4 days before euthanasia to recruit macrophages to the peritoneal cavity. After euthanasia, the epidermal layer that covers the abdomen was carefully cut and 6 ml of cold Dulbecco’s Phosphate Buffered Saline solution (Sigma-Aldrich, D8537) was injected into the peritoneal cavity. About 4 ml of intraperitoneal content was collected and centrifuged at 400 × g, 4°C for 5 min. The supernatant was discarded, and the pelleted cells were resuspended in Roswell Park Memorial Institute medium (RPMI-1640, Vitrocell, 9) containing 10% (*v/v*) fetal bovine serum (Vitrocell, 00011), 1% (*v/v*) Antibiotic-Antimycotic 100X (Thermo Fisher Scientific, 15240062), 2 mM L-glutamine, 17.9 mM sodium bicarbonate, and 1 mM sodium pyruvate. Cell counting was performed in a hemocytometer. Cells were seeded in a 24-well plate (Nest Biotechnology, 702001) or 96-well plate (Greiner Bio-one, 655090) and allowed to adhere for 2 h. Subsequently, non-adherent cells were washed away with Dulbecco’s Phosphate Buffered Saline solution (Sigma-Aldrich, D8537) and peritoneal macrophages were incubated in complete fresh RPMI-1640 medium at 37°C, 5% (*v/v*) CO_2_ during 72 h before experiments.

### Proximity Ligation Assay

BMDM and PM were seeded in a 96-well plate with a µClear® bottom (Greiner Bio-one, 655090) at 60,000 cells/well, 24 h before assays. Cells were washed five times with phosphate-buffered saline solution (PBS, 137 mM NaCl, 2.7 mM KCl, 4.3 mM NaH_2_PO_4_, 1.4 mM KH_2_PO_4_, pH 7.2) and fixed with 3.7% (*v/v*) formaldehyde (Dinâmica, 60READIN001948, São Paulo, Brazil) diluted in PBS for 10 min at room temperature. Next, cells were washed three times with wash buffer 1% (*w/v*) bovine serum albumin (BSA, Amresco, 332, Albany, NY) and 0.02% (*v/v*) Triton X-100 (Sigma-Aldrich, T8787) in PBS and permeabilized with 0.5% (*v/v*) Triton X-100 in PBS for 10 min. Cells were washed three times with washing buffer, and the free aldehydes were blocked using 10 mM glycine (Synth, 2,689, Diadema, Brazil) in PBS for 5 min. Cells were washed two times, and unspecific sites were blocked using 3% (*w/v*) BSA and 0.02% (*v/v*) Triton X-100 in PBS for 30 min (Assis et al., 2017). Cells were washed once again and incubated with 0.5 µg/ml mouse anti-Vdac1 clone 20B12AF2 (Abcam, ab14734, Cambridge, United Kingdom) and 0.65 µg/ml rabbit anti-Ip3r1 (Abcam, ab5804) antibodies overnight at 4°C. Cells were washed three times, and proximity ligation assay (PLA) was performed using Duolink® *In Situ* PLA Probe Anti-Mouse Minus (Sigma-Aldrich, DUO92004), Duolink® *In Situ* PLA Probe Anti-Rabbit Plus (Sigma-Aldrich, DUO92002), and Duolink® *In Situ* Detection Reagent Far-Red (Sigma-Aldrich, DUO92013) according to the manufacturer’s protocol. Finally, cells were incubated with 5 µg/ml DAPI (Thermo Fisher Scientific, 62248, Waltham, MA) diluted in PBS for 15 min. The strategy to detect Ip3r1-Vdac1 interaction was reported previously (Tubbs et al., 2014), allowing the detection of the ER and mitochondrial membranes interacting closer than 40 nm (Fredriksson et al., 2002; Söderberg et al., 2006). Cells were maintained in 200 μL of PBS before imaging in an automated fluorescence microscope.

### Mitochondria Staining

BMDM and PM were seeded at 60,000 cells/well in a 96-well plate with a µClear® bottom (Greiner Bio-one, 655090) and cultured as previously described. Cells were incubated with 250 nM MitoTracker® Red CMXRos (Thermo Fisher Scientific, M7512) and 5 µg/ml Hoechst 33342 (Thermo Fisher Scientific, H3570) in RPMI-1640 medium (Vitrocell, 9) in the absence of fetal bovine serum, for 45 min, at 37°C and 5% (*v/v*) CO_2_. Cells were kept in 200 μL of PBS solution before imaging.

### Preparation of Oxidized Low-Density Lipoproteins

LDL was isolated from human plasma using KBr-density gradient as previously described (Havel et al., 1955). Briefly, plasma was isolated from blood by centrifugation at 2,000 × g, 4°C for 10 min. Plasma density was adjusted to 1.100 g/ml using specific gravity KBr Solution A (density at 1.006 g/ml) and centrifuged at 17,136 × g, 4°C for 18 h using a 70.1 Ti fixed-angle titanium rotor (Beckman Coulter, 342184, Brea, CA) and Optma LE-8OK Ultracentrifuge (Beckman Coulter, 8043-30-1192). The supernatant fraction containing very-low-density lipoprotein (VLDL) was discarded. Solution density was adjusted using specific gravity KBr Solution B (density at 1,182 g/ml) and centrifuged at 17,136 × g, 4°C for 24 h. The supernatant fraction (orange ring) containing low-density lipoprotein (LDL) was isolated and further dialyzed in 0.85% (w/v) NaCl at 4°C for 24 h. LDL was incubated with 10 μM CuSO_4_ at 37°C for 24 h. Oxidized LDL (ox-LDL) was dialyzed in 0.85% (w/v) NaCl at 4°C for 24 h. Protein content was determined using Pierce^TM^ 660 nm Protein Assay reagent (Thermo Fisher Scientific, 22660) and Pierce^TM^ Bovine Serum Albumin Standard Pre-diluted Set (Thermo Fisher Scientific, 23208) as standards.

### Foam Cell Formation Assay

BMDM and PM were resuspended in the respective complete culture medium as described and seeded at 200,000 cells/well in a µClear® bottom (Greiner Bio-one, 655090). Cells were incubated at 37°C, 5% (v/v) CO_2_ for 2 days (BMDM) and 3 days (PM). The culture medium was replaced by a fresh one containing only 3% (v/v) fetal bovine serum (Vitrocell, 11). Cells were incubated at 37°C, 5% (v/v) CO_2_ for 4 h. Following, cells were incubated in a culture medium containing 1% (v/v) fetal bovine serum (Vitrocell, 11) and ox-LDL (equivalent to 100 μg/ml of cholesterol). Cells were incubated at 37°C, 5% (v/v) CO_2_ for 24 h. Next, cells were washed five times with PBS and fixed with 3.7% (v/v) formaldehyde (Dinâmica, 60READIN001948) diluted in PBS for 10 min at room temperature. Cells were washed five times with PBS. Neutral lipids were stained with Oil Red O (ORO, Sigma-Aldrich, 625) as described (Xu et al., 2010), while nuclei were stained with Hoechst 33342 (Thermo Fisher Scientific, H3570). Cells were maintained in 200 μL of PBS before imaging in an automated fluorescence microscope.

### Fluorescence Microscopy Using a High-Content Imaging System

BMDM and PM cultured in a 96-well plate with a µClear® bottom (Greiner Bio-one, 655090) and labeled with fluorescent dyes were imaged in the ImageXpress® Micro Confocal High Content Imaging System (Molecular Devices, Sunnyvale, CA). Proximity ligation assay and superoxide anion images were obtained using CFI Super Plan Fluor ELWD 40X/0.60 (Nikon, MRH08430, Melville, NY) with 0.17 correction collar. Mitochondrial network and lipid droplets were imaged using the CFI Plan Apochromat Lambda 60X/0.95 Dry Objective Lens (Nikon, MRD00605) with 0.17 correction collar. Foam cells were imaged using CFI Plan Apochromat Lambda 10X/0.45 (Nikon, MRD00105).

### Endoplasmic Reticulum–Mitochondria Interaction Analysis

Fluorescence images of cells stained with Cyanine 5 (for PLA), DAPI, or Hoechst 33342 (for nuclei) were analyzed in the MetaXpress® 6 software (Molecular Devices) using the Trans-fluor tool (Molecular Devices). PLA fluorescent dots and nuclei were identified based on shape and fluorescence intensity above the background. Results were expressed as the number of PLA fluorescent dots per cell, which was corrected by the respective value found in negative controls (PLA performed in cells which were not incubated with anti-Vdac1 or anti-Ip3r1 antibodies). Representative images were corrected regarding intensity above background, contrast, and brightness using the software Fiji (Schindelin et al., 2012).

### Mitochondrial Network Morphology Analysis

A customized script was developed using MetaXpress® 6 software (Molecular Devices) in order to quickly and automatically identify and characterize the morphological parameters of mitochondria imaged in the high-content imaging system. All images were analyzed as follows: 1) nuclei raw images were first analyzed using the “Finding Rounding Objects” tool, allowing identifying small and symmetrically round objects based on size and intensity above the background criteria; 2) next, we applied the “Grow Objects Without Touching” tool to expand the previously identified nuclei, but without touching each other, creating an artificial cytoplasm perimeter; 3) mitochondria raw images were background-subtracted using the “Top Hat” tool, where small bright spots were identified based on the shape and size of filters; and 4) we applied the “Find Blobs” tool on the last image, using size and intensity above the background criteria to identify irregularly shaped objects, i.e., mitochondria. This last tool does not separate objects that are touching each other, so it is useful to identify elongated or branched mitochondria. Each identified mitochondrion was characterized based on parameters such as length, breadth, area, and perimeter. Results were expressed as average length, breadth, area, and perimeter per imaged field in fluorescence microscopy. The aspect ratio (organelle elongation) was calculated by the ratio between the major and minor axis from each individual mitochondrion. On the other hand, the form factor was calculated by the equation (perimeter^2^)/(4π*area), which represents the branching organelle degree (Trudeau et al., 2011). Representative images were corrected regarding intensity above the background, contrast, and brightness using the software Fiji (Schindelin et al., 2012).

### Lipid Droplets’ Size and Density

A customized script was developed using MetaXpress® 6 software (Molecular Devices) to identify and characterize lipid droplets’ size and number in foam cells. All images were analyzed as follows: 1) nuclei raw images were first analyzed using the “Finding Rounding Objects” tool, allowing the identification of small and symmetrically round objects based on size and intensity above the background criteria; 2) next, we applied the “Grow Objects Without Touching” tool, to expand the previously identified nuclei, but without touching each other, creating an artificial cytoplasm perimeter; 3) Oil Red O (ORO) raw images were background-subtracted using the “Top Hat” tool, where small bright spots were identified based on the shape and size of filters; and 4) we applied the “Find Blobs” tool on the last image, using size and intensity above the background criteria to identify lipid droplets. Each identified lipid droplet was characterized based on its size. Results were expressed as lipid droplet average size or lipid droplet number per cell in each imaged field in fluorescence microscopy. Representative images were corrected regarding intensity above the background, contrast, and brightness using the software Fiji (Schindelin et al., 2012).

### Mitochondrial Bioenergetics

BMDM and PM were seeded at a density of 250,000 cells/well in the Seahorse XF24 Flux Cell Culture Micro Plate (Agilent Technologies, 102340-100, Santa Clara, CA). Cells were incubated with a fresh non-buffered medium in the absence of phenol red and fetal bovine serum, at 37°C, for 60 min, with no CO_2_ before loading into a Seahorse XFp Extracellular Flux Analyzer (Agilent Technologies). Different drugs were added to the cell medium to assess the oxygen consumption rates (OCRs) associated with different mitochondrial states. This includes 1 µM oligomycin (ATP synthase inhibitor), 1 µM carbonyl cyanide 4-(trifluoromethoxy) phenylhydrazone (FCCP, uncoupler), and a mix of 1 µM antimycin A (complex III inhibitor) plus 1 µM rotenone (complex I inhibitor). The oxygen consumption parameters’ calculations were as follows: 1) non-mitochondrial oxygen consumption: the minimum rate measurement after rotenone and antimycin injection; 2) basal respiration: (last rate measurement before the oligomycin injection)–(non-mitochondrial oxygen consumption rate); 3) maximal respiration: (maximal rate measurement after FCCP injection)–(non-mitochondrial oxygen consumption rate); 4) proton leak: (minimal rate measurement after oligomycin injection)–(non-mitochondrial oxygen consumption rate]; and 5) ATP-linked respiration (last rate measurement before the oligomycin injection)–(minimal rate measurement after oligomycin injection). The extracellular acidification rate (ECAR) parameters measured in this experiment calculations were as follows: 1) glycolysis: last measurement before oligomycin injection; 2) glycolytic capacity: maximal rate measurement after oligomycin injection.

### Generation of Superoxide Anion

BMDM and PM were seeded in a 96-well plate with a µClear® bottom (Greiner Bio-one, 655090) at the density of 50,000 cells/well and incubated at 37°C, 5% (*v/v*) CO_2_ for 24 h (BMDM) or 72 h (PM) before experiments. The cellular medium was replaced, and cells were washed once with PBS at 37°C. Then, cells were incubated with 2 μM Dihydroethidium (DHE, Thermo Fisher Scientific, D1168) or 10 μM MitoSOX^TM^ Red Mitochondrial Superoxide Indicator (Thermo Fisher Scientific, M36008), both diluted in PBS solution, at 37°C, 5% (*v/v*) CO_2_ for 10 min. Next, cells were incubated with 5 μg/ml Hoechst 33342 (Thermo Fisher Scientific, H3570) in PBS solution for 15 min. Cells were fixed with 3.7% (*v/v*) formaldehyde (Dinâmica, 60READIN001948) diluted in PBS for 10 min at room temperature. Cells were washed three times with PBS. Cells were kept in 200 μL PBS solution before imaging. All samples were background-subtracted using non-stained cells.

### Hydrogen Peroxide (H_2_O_2_) Release

BMDM and PM were seeded at a density of 250,000 cells/well in a 96-well plate with a µClear® bottom (Greiner Bio-one, 655090). The hydrogen peroxide (H_2_O_2_) released was quantified using Amplex® Red Hydrogen Peroxide/Peroxidase Assay (Thermo Fisher Scientific, A22188). Experiments were carried out in 100 µL of PBS pH 7.4 containing 11.1 mM glucose, 25 μM Amplex® Red Reagent (Thermo Fisher Scientific, A12222), and 0.2 U/ml Pierce^TM^ Horseradish Peroxidase (HRP, Thermo Fisher Scientific, 31490) in the dark at 37°C for 40 min. The resorufin product formed was monitored by fluorescence spectroscopy (excitation energy at 530 nm and emission energy at 590 nm) every 10 min in Spectramax M3 (Molecular Devices). Background signal was determined by adding 500 U/ml Catalase from Bovine Liver (Sigma-Aldrich, E3289) in one well to convert hydrogen peroxide into water, abolishing the non-specific signal. Cells were incubated with 1 µM FCCP to eliminate the mitochondrial contribution to H_2_O_2_ generation. Thus, the mitochondrial H_2_O_2_ released was calculated by the difference between the total H_2_O_2_ released and FCCP-treated cells. Fluorescence data were converted into H_2_O_2_ concentration using a standard curve with known H_2_O_2_ concentrations. H_2_O_2_ released rates (H_2_O_2_ ηM/min) were further normalized by the amount of DNA in each sample after staining cells with violet crystal.

### Reduced (GSH) and Oxidized (GSSG) Glutathione

BMDM and PM were plated at 1,000,000 cells/well in a 6-well plate (Nest Biotechnology, 703001) and allowed to rest for 24 and 72 h, respectively. Next, adhered cells were harvested and incubated with 300 μL RIPA Buffer (Sigma-Aldrich, R0278) supplemented with cOmplete^TM^ Protease Inhibitor Cocktail Tablets (Roche, 11697498001) for 15 min at 4°C. The cell lysate was centrifuged at 10,000 × g, 4°C for 15 min, and the supernatant was collected for further analyses. Total reduced glutathione (GSH) content was assayed in BMDM and PM lysates using the Glutathione Assay kit (Cayman Chemical, 703002, Ann Arbor, MI). This assay is based on the reaction between the sulfhydryl groups of GSH with 5,5′-dithio-bis-2-(nitrobenzoic acid) (DTNB), yielding a soluble yellowish product called 5-thio-2-nitrobenzoic acid (TNB), which can be monitored by spectroscopy. To determine oxidized glutathione (GSSG) content apart from GSH, we previously treated samples and standards with 32 mM 2-vinylpiridine (Sigma-Aldrich, 132292) for 60 min at room temperature. Total GSH and GSSG levels were determined kinetically in SpectraMax M3 (Molecular Devices) by measuring the absorbance at 409 nm, for 25 min at 26°C. Results were normalized based on the protein content in the cell lysate using Pierce^TM^ 660 nm Protein Assay reagent (Thermo Fisher Scientific, 22660) and Pierce^TM^ Bovine Serum Albumin Standard Pre-Diluted Set (Thermo Fisher Scientific, 23208) as standards.

### Real-Time Quantitative Polymerase Chain Reaction

BMDM and PM were seeded at 150,000 cells/well in a 24-well Cell Culture Plate (Nest Biotechnology, 702001). Total RNA was extracted using TRIzol^TM^ reagent (Thermo Fisher Scientific, 15596026) according to the manufacturer’s protocol and then quantified in a NanoDrop^TM^ 2000/2000c spectrophotometer (Thermo Fisher Scientific). The amount of 2 µg of total RNA was reverse transcribed using a High-Capacity cDNA Reverse Transcription Kit (Thermo Fisher Scientific, 4368814). The amplification step was carried out using Fast SYBR^TM^ Green Master Mix (Thermo Fisher Scientific, 4385612), 50 ng of each cDNA, and 300 nM of each forward and reverse oligonucleotide (Supplementary Table S1). RT-qPCR assays were carried out in the 7,500 Real-Time PCR System (Applied Biosystems, Foster City, CA). Genes involved in mitochondrial dynamics were normalized based on the Actb gene expression (Chung et al., 2017). Genes involved in inflammation were normalized based on the Rplp0 gene expression. Relative abundance of mRNA was quantified using the threshold cycle method (ΔΔC_T_) (Livak and Schmittgen, 2001).

### Macrophage Phagocytosis

BMDM and PM were resuspended in a complete culture medium as previously described and seeded at 200,000 cells/well in a 96-well plate (Nest Biotechnology, 701001) in two plates. Cells were incubated at 37°C, 5% (v/v) CO_2_ for 24 (BMDM) and 72 h (PM). Phagocytosis assay was carried out as previously described (de Lima et al., 2008) with slight modifications. Briefly, cells were incubated with 1 × 10^6^ particles of Zymosan A from *Saccharomyces cerevisiae* (Sigma-Aldrich, Z4250) stained with Neutral Red Reagent (Sigma-Aldrich, N4638) in a complete culture medium at 5% (v/v) CO_2_ and 37°C for 30 min. Next, the supernatant was removed and cells were fixed with Baker’s solution (4% (v/v) formaldehyde, 2% (w/v) sodium chloride, and 1% (w/v) calcium acetate) at 37°C for 30 min. After that, the plate was centrifuged at room temperature at 370 × g for 5 min and washed twice with PBS. Finally, cells were incubated with 100 μl/well of acidified alcohol solution (10% (v/v) acetic acid and 40% (v/v) ethanol solution) at 37°C for 30 min. Neutral red staining was measured by absorbance at 550 nm in a SpectraMax M3 device (Molecular Devices). Adhesion assay was performed on a second plate. Cells were washed three times with PBS, and the adhered cells were fixed with 50% (v/v) methanol for 10 min at room temperature. Next, cells were stained with 0.5% (w/v) Giemsa solution (Sigma-Aldrich, G5637) for 40 min at room temperature and further washed with PBS. The remaining dye was solubilized with 50% (v/v) methanol for 30 min at room temperature. Giemsa staining absorbance was determined spectrophotometrically at 550 nm in a SpectraMax M3 device (Molecular Devices). Data were presented as phagocytosis by adhered cells.

### Cytokine Secretion Assay

BMDM and PM were seeded at 300,000 cells/well in a 24-well plate (Nest Biotechnology, 702001). Interleukin-1β was quantified in the culture medium using the Mouse Il-1β Uncoated ELISA kit (Thermo Fisher Scientific, 88-7013) according to the manufacturer’s protocol. For normalization, adhered cells were harvested and incubated with 100 µL RIPA buffer (Sigma-Aldrich, R0278) containing cOmplete^TM^ Protease Inhibitor Cocktail (Roche, 11697498001) for 15 min at 4°C. The cellular lysate was centrifuged at 18,000 × g, 4°C for 15 min. The protein content was determined in the supernatant using Pierce^TM^ 660 nm Protein Assay reagent (Thermo Fisher Scientific, 22660, Rockford, IL) and Pierce^TM^ Bovine Serum Albumin Standard Pre-Diluted Set (Thermo Fisher Scientific, 23208) as standards. Absorbance was measured in a SpectraMax M3 spectrophotometer (Molecular Devices) at the wavelength of 660 nm. Results were expressed as interleukin-1β amount by total cell protein.

### Statistical Analyses

Data were expressed as mean ± standard error (SE). Statistical analyses were carried out with the software GraphPad Prism 7 (GraphPad). Data were compared using two-tailed unpaired Student’s t-test when datasets passed the D'Agostino–Pearson or Saphiro–Wilk omnibus normality test (alpha = 0.05). Otherwise, data were compared using a two-tailed unpaired non-parametric Mann–Whitney test with 95% confidence. Statistical significance was defined as a value of *p* < 0.05. Non-significant differences between groups were not displayed in the panels.

## Results

We tested the effects of hypercholesterolemia by comparing macrophages of C57BL/6J wild-type (WT) and LDL receptor knockout (LDLr^−/−^) mice. Then, we tested the effects of pravastatin by comparing macrophages from treated (prava) and non-treated (control) LDLr^−/−^ mice. We used two macrophage sources: bone marrow-derived macrophages (BMDM) and thioglycolate-elicited peritoneal macrophages (PM). The results of the latter are shown as supplementary material.

### Hypercholesterolemia Induces Increases in ER–mitochondria Interaction and Mitochondrial Network Branching in Macrophages

To evaluate ER–mitochondria interaction, we determined the number of physical contact sites at the MAM, using the protein pair IP3 receptor (Ip3r1) and voltage-dependent anion selective channel (Vdac1) at the ER and mitochondria interfaces, respectively.

**FIGURE 1:**
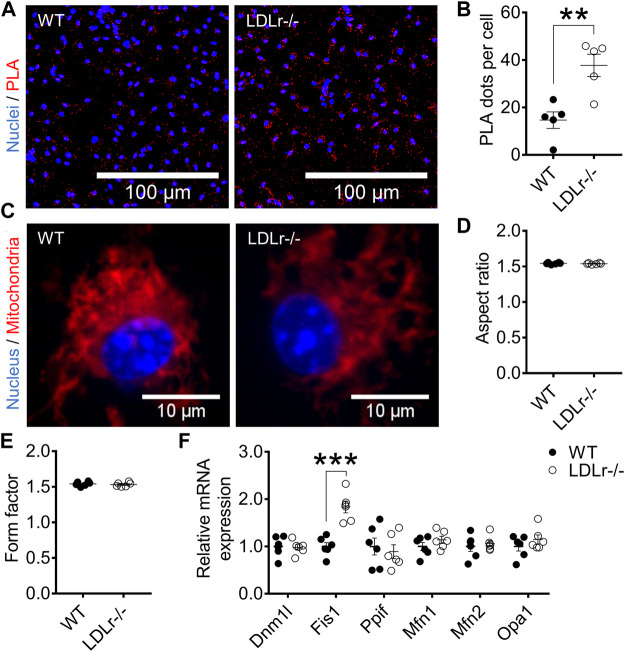
Hypercholesterolemia increases ER–mitochondria interaction and Fis1 gene expression in BMDM. **(A)** Representative proximity ligation assay (PLA) images at 40× magnification and **(B)** quantitative analysis of Ip3r1-Vdac1 interactions in BMDM from WT and LDLr^−/−^ mice. Ip3r1-Vdac1 interactions detected through PLA assay were labeled with Cyanine 5 (red) and nuclei with DAPI (blue). Two replicates per mouse were considered, each corresponding to the average of nine fields analyzed by fluorescence microscopy. WT: *n* = 5 mice and LDLr^−/−^: *n* = 5 mice. **(C)** Representative images of mitochondria network at 60× magnification and quantitative analysis of mitochondria aspect ratio **(D)** and form factor **(E)**. Four replicates per mouse were considered, each corresponding to the average of nine fields analyzed by fluorescence microscopy. Mitochondria were stained with MitoTracker (red) and nuclei with Hoechst 33342 (blue). WT: *n* = 6 mice and LDLr^−/−^: *n* = 6 mice. **(F)** Relative expression of genes involved in mitochondrial fusion and fission. WT: *n* = 6 mice and LDLr^−/−^: *n* = 6 mice. Data are expressed as mean ± SE. Statistical analyses were performed using two-tailed unpaired Student’s t-test. ** and *** represent *p* < 0.01 and 0.001, respectively.

BMDM of LDLr^−/−^ mice presented a marked 2.6-fold increase in ER-mitochondria contact sites when compared with cells of WT mice (Figures 1A,B). No significant changes in mitochondrial network morphology in BMDM of WT and LDLr^−/−^ were observed concerning the aspect ratio (indicator of mitochondria elongation) and form factor (indicator of mitochondria branching) degrees (Figures 1C–E).

Mitochondrial morphology reflects the balance between mitochondria fusion and fission. Fusion is performed by mitofusin-1 (Mfn1), mitofusin-2 (Mfn2), and optic atrophy 1 (Opa1), whereas fission depends on dynamin-related protein 1 (Dnm1l), peptidylprolyl isomerase F (Ppif, also called cyclophilin D), and fission-1 (Fis1). BMDM of LDLr^−/−^ mice showed a 1.8-fold increase in Fis1 gene expression when compared with cells of WT mice (Figure 1F). These results suggest hypercholesterolemia favors mitochondria fragmentation in BMDM, an aspect not evaluated in the previous mitochondrial network analysis.

Regarding peritoneal macrophages (PM), different from BMDM, we did not see significant differences in the number of contact sites at the MAM between both groups (Supplementary Figures S1A,B). PM mitochondrial network elongation (aspect ratio) was not altered, but the branching (form factor) was significantly increased (∼5%) in LDLr^−/−^ PM when compared with WT mice (Supplementary Figures S1C–E). In addition, PM of WT and LDLr^−/−^ mice showed similar gene expression of mitochondrial fusion and fission markers (Supplementary Figure S1F).

Therefore, the main effects of hypercholesterolemia are induction of ER-mitochondria contact sites at the MAM in BMDM and a more branched mitochondrial network in PM.

### Hypercholesterolemia Does Not Influence Mitochondrial Respiratory Rates but Modulates Oxidant Generation in Macrophages

Mitochondrial oxygen consumption rates (OCR) in several conditions (basal, phosphorylating, resting, and stimulated) and the extracellular acidification rates (ECAR), indicative of glycolysis, were not different in macrophages of WT and LDLr^−/−^ mice, neither in BMDM (Supplementary Figures S2A–I) nor in PM (Supplementary Figures S3A–I). Thus, hypercholesterolemia does not affect mitochondrial respiration and glycolysis in macrophages measured after 3 (PM) or 7 (BMDM) days of culture.

**FIGURE 2:**
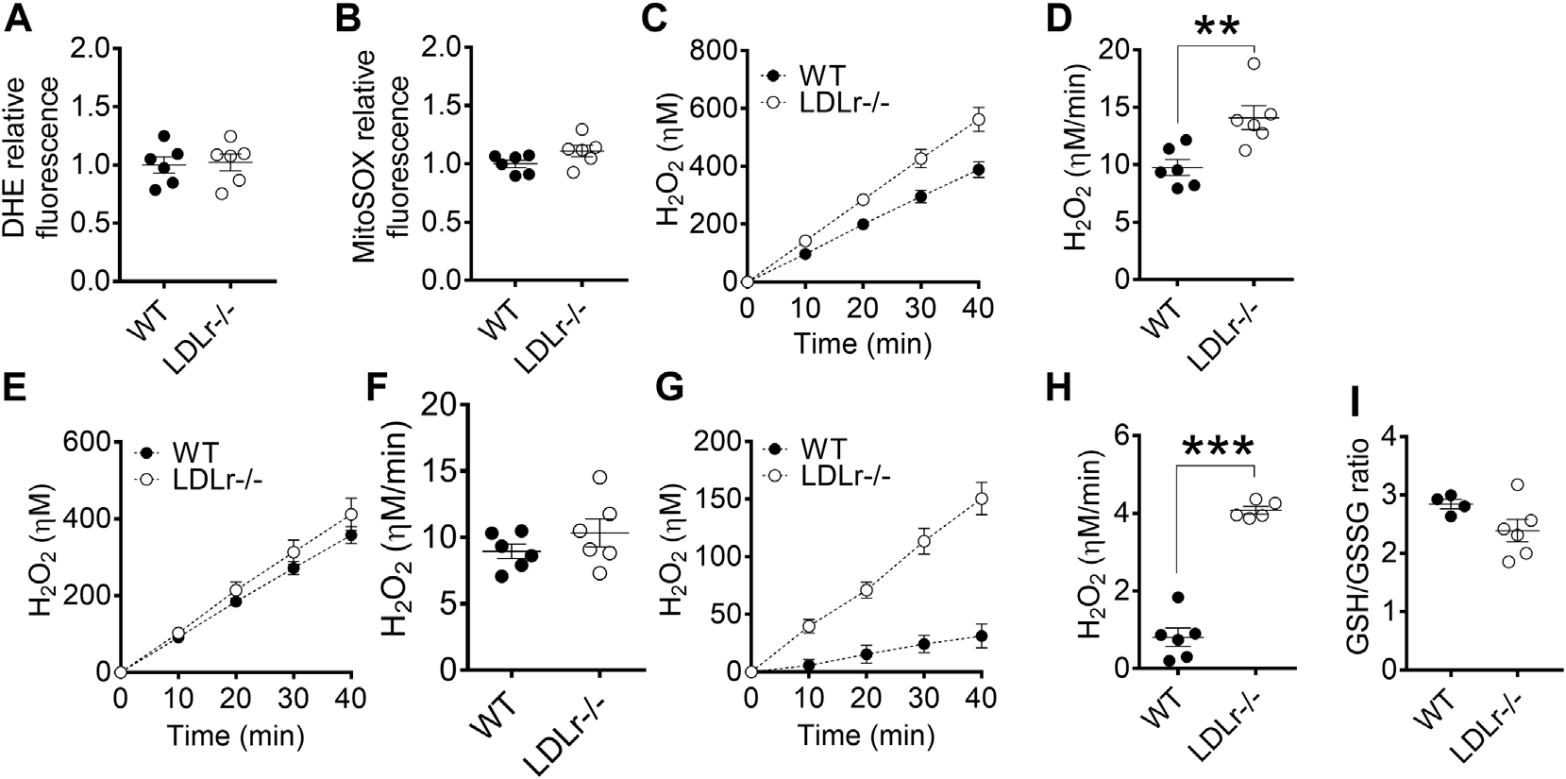
Hypercholesterolemia increases mitochondrial hydrogen peroxide release in BMDM. Detection of global **(A)** and mitochondria-derived **(B)** superoxide anion production in BMDM from WT and LDLr^−/−^ mice. Four replicates per mouse were considered, each corresponding to the average of nine fields analyzed by fluorescence microscopy. WT: *n* = 6 mice and LDLr^−/−^: *n* = 6 mice. Average curves and rate quantitation of total **(C,D)**, non-mitochondrial **(E,F)**, and mitochondrial **(G,H)** release of hydrogen peroxide (H_2_O_2_). Values were normalized by DNA amount in each well. Three replicates per mouse were considered. Data are expressed as mean ± SE. WT: *n* = 6 mice and LDLr^−/−^: *n* = 6 mice. **(I)** Oxidative stress was assessed by GSH/GSSG ratio. WT: *n* = 4 mice and LDLr^−/−^: *n* = 6 mice. Statistical analyses were performed using two-tailed unpaired Student’s t-test. ** and *** represent *p* < 0.01 and 0.001, respectively.

Next, we evaluated superoxide anion production (probed with DHE and MitoSOX) and hydrogen peroxide release (probed with Amplex red) in macrophages. We verified in BMDM no significant differences in global (Figure 2A) and mitochondria-derived (Figure 2B) superoxide anion production between groups. However, PM of LDLr^−/−^ mice presented a 10% decrease in overall cell superoxide anion production (Supplementary Figure S4A), while the pool of superoxide derived from mitochondria (Supplementary Figure S4B) remained unaltered when compared with PM of WT mice.

Superoxide anion is rapidly converted into hydrogen peroxide (H_2_O_2_) in most cells. The rate of total cell H_2_O_2_ released in BMDM of LDLr^−/−^ mice was increased by 44% when compared with cells of WT mice (Figures 2C,D), with no significant contribution of non-mitochondrial sources (Figures 2E,F), but marked increase in the release of H_2_O_2_ coming from the mitochondria of LDLr^−/−^ mice (Figures 2G,H). GSH/GSSG ratio remained unaltered in BMDM of WT and LDLr^−/−^ mice (Figure 2I). The rate of total cell H_2_O_2_ released in PM of LDLr^−/−^ mice was 22.6% higher than in cells of WT mice (Supplementary Figures S4C,D). Unlike in BMDM, we observed an increase in hydrogen peroxide released by non-mitochondrial sources (Supplementary Figures S4E,F) and decreased mitochondrial-derived H_2_O_2_ (Supplementary Figures S4G,H). Despite the changes in oxidant generation, we did not see any alteration in GSH/GSSG ratio between PM from WT and LDLr^−/−^ groups (Supplementary Figure S4I).

Thus, both BMDM and PM of LDLr^−/−^ showed significant increases in H_2_O_2_ generation rates, although the sources of this oxidant (global vs. mitochondrial) may differ in each macrophage type.

### Hypercholesterolemia Enhances Foam Cell Formation and Phagocytosis in Bone Marrow-Derived Macrophages

Macrophages become foam cells as they take up chemically modified LDL such as ox-LDL (Goldstein and Brown, 2015). We evaluated foam cell formation by staining cellular global lipids with Oil Red O (ORO) and determined lipid droplets’ size and density in cells after incubation with ox-LDL.

**FIGURE 3:**
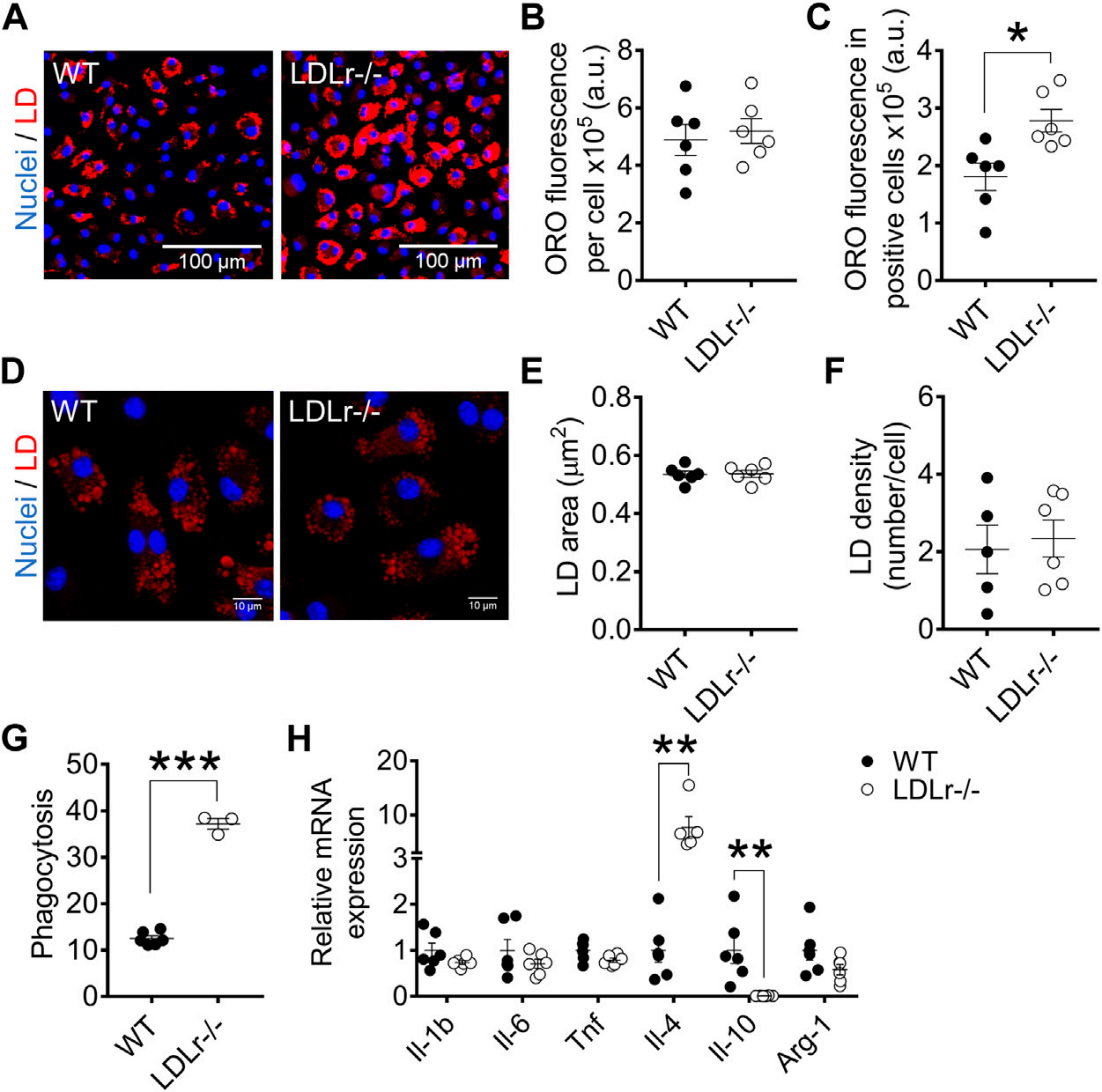
Hypercholesterolemia increases foam cell formation and phagocytosis and modulates anti-inflammatory gene expression in BMDM. **(A)** Representative images at 10× magnification of BMDM from WT and LDLr^−/−^ mice after incubation with ox-LDL. Neutral lipids were stained with ORO fluorescent dye (red) and nuclei with Hoechst33342 (blue). Quantitative analysis of ORO fluorescence intensity in all cells **(B)** and in positive cells **(C)**. **(D)** Representative images of lipid droplets at 60× magnification and stained with ORO (red) and nuclei with Hoechst33342 (blue). Quantitative analysis of lipid droplets’ size **(E)** and density **(F)** from images displayed in **(D)**. Three replicates per mouse were considered, each corresponding to the average of nine fields analyzed by fluorescence microscopy. WT: *n* = 6 mice and LDLr^−/−^: *n* = 6 mice. **(G)** Zymosan phagocytosis. Two replicates per mouse were considered. WT: *n* = 6 mice and LDLr^−/−^: *n* = 3 mice. **(H)** Relative mRNA expression of genes involved in inflammation. WT: *n* = 6 mice and LDLr^−/−^: *n* = 6 mice, with two replicates for each gene. Data are expressed as mean ± SE. Statistical analyses were performed using two-tailed unpaired Student’s t-test. *, **, and *** represent *p* < 0.05, 0.01, and 0.001, respectively.

In BMDM, global ORO fluorescence was similar in WT and LDLr^−/−^ mice (Figures 3A,B). Because not all cells become foam cells after incubation with ox-LDL, we counted macrophages containing lipid droplets, which we called “LD-positive cells,” and measured ORO fluorescence in these positive cells. ORO fluorescence in LD-positive cells of LDLr^−/−^ mice was indeed significantly higher when compared with cells of WT mice (Figure 3C). Lipid droplet area (Figures 3D,E) and density (Figure 3F) were equivalent in macrophages of WT and LDLr^−/−^ groups. In addition, BMDM of LDLr^−/−^ mice presented a marked 3-fold increase in phagocytosis when compared to macrophages of WT mice (Figure 3G). Thus, genetic endogenous hypercholesterolemia induces exacerbation of ox-LDL-mediated foam cell formation and phagocytic activity of BMDM.

In PM, global ORO fluorescence (Supplementary Figures S5A,B), ORO fluorescence in LD-positive cells (Supplementary Figure S5C), lipid droplet area (Supplementary Figures S5D,E), and density (Supplementary Figure S5F) were similar in PM of WT and LDLr^−/−^ mice. Phagocytic activity did not differ in both groups (Supplementary Figure S5G). Therefore, hypercholesterolemia did not affect foam cell formation and phagocytosis in PM.

### Hypercholesterolemia Modulates Macrophage Inflammatory Gene Expression

We investigated whether hypercholesterolemia could affect the macrophage inflammation-related gene expression profile. BMDM of LDLr^−/−^ mice presented a 6.5-fold increase in interleukin-4 (Il-4) and a 99% decrease in interleukin-10 (Il-10) mRNA levels compared with WT cells. The gene expression of interleukin-1 β (Il-1b), tumor necrosis factor (Tnf), interleukin-6 (Il-6), and arginase-1 (Arg-1) remained unaltered in LDLr^−/−^ BMDM (Figure 3H).

PM of LDLr^−/−^ mice presented altered gene expression of either pro- and anti-inflammatory markers when compared with PM of WT mice. This change includes an 89% decrease in Il-6, a 5.0-fold increase in Il-10, and a 56% decrease in Arg-1 levels. Il-1b, Tnf and Il-4 levels were not affected in PM (Supplementary Figures S5H).

Therefore, hypercholesterolemia modulates either pro- and anti-inflammatory genes in PM, while only anti-inflammatory genes are affected in BMDM.

### Pravastatin Treatment Reduces ER–mitochondria Interaction and Enhances Mitochondrial Branching and Dynamics-related Gene Expression in Macrophages

Considering statins are drugs used to reduce cholesterol levels in the plasma, we hypothesized they could counteract, at least partially, the altered responses of macrophages to hypercholesterolemia. Thus, we treated LDLr^−/−^ mice for 3 months with pravastatin (prava) dissolved in drinking water and compared their macrophages with those of non-treated (control) LDLr^−/−^ mice.

**FIGURE 4:**
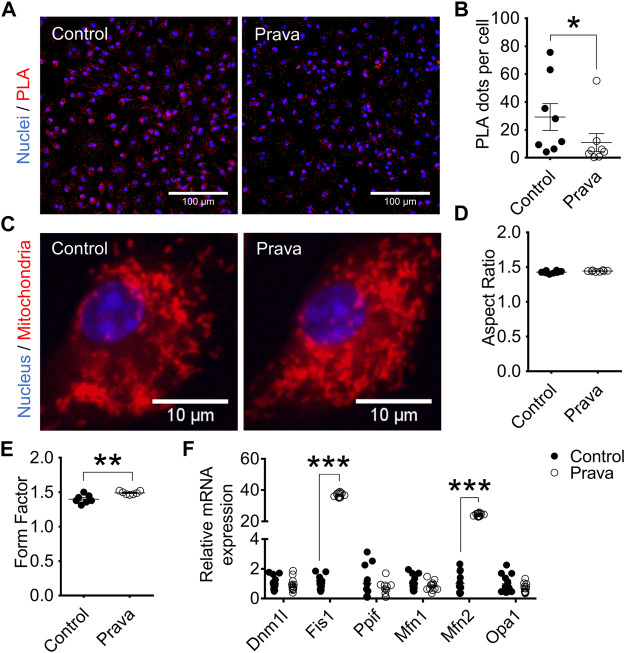
Pravastatin reduces ER–mitochondria interaction, increases mitochondrial branching, and upregulates Fis1 and Mfn2 gene expression in BMDM. **(A)** Representative PLA images at 40× magnification and **(B)** quantitative analysis of Ip3r1-Vdac1 interaction in BMDM from non-treated (control) and pravastatin-treated (prava) LDLr^−/−^ mice. Two replicates per mouse were considered, each corresponding to the average of nine fields analyzed by fluorescence microscopy. Control: *n* = 8 mice and prava: *n* = 8 mice. Statistical analyses were performed using a two-tailed nonparametric Mann–Whitney test. **(C)** Representative images of the mitochondrial network at 60× magnification and quantitative analysis of mitochondria aspect ratio **(D)** and form factor **(E)**. Four replicates per mouse were considered, each corresponding to the average of nine fields analyzed by fluorescence microscopy. Data are expressed as mean ± SE. Control: *n* = 8 mice and prava: *n* = 8 mice for aspect ratio analysis, and control: *n* = 7 mice and prava: *n* = 7 mice for form factor analysis. **(F)** Relative gene expression of mitochondrial fusion and fission markers in BMDM. Control: *n* = 13 mice and prava: *n* = 12 mice. Data are expressed as mean ± SE. Statistical analyses were performed using a two-tailed unpaired Student’s t-test. *, **, and *** represent *p* < 0.05, 0.01, and 0.001, respectively.

In BMDM, pravastatin treatment decreased the number of sites (63%) of ER–mitochondria physical interactions (Figures 4A,B). In addition, pravastatin treatment of LDLr^−/−^ mice had no effects on the mitochondrial elongation indicator (aspect ratio), but increased mitochondrial branching (form factor) (Figures 4C–E). Similar to BMDM, PM of pravastatin-treated LDLr^−/−^ mice had less sites of physical interaction between the ER and mitochondria (∼60%) when compared to PM of untreated control LDLr^−/−^ mice (Supplementary Figures S6A,B). Pravastatin induced no significant changes in the mitochondria network morphology of PM (Supplementary Figures S6C–E). However, considering control plus prava data, we noticed an inverse correlation between the number of ER-mitochondria contact sites and the mitochondrial branching (form factor) (Supplementary Figure S6F,G). Thus, these results show chronic pravastatin treatment reduces ER–mitochondria interaction in both types of macrophages and induces a more branched mitochondrial network in BMDM.

We also analyzed genes involved in mitochondria fusion and fission dynamics. In BMDM, Fis1 and Mfn2 genes were markedly upregulated, 37-fold and 24-fold, respectively. In contrast, Dnm1l, Ppif, Mfn1, and Opa1 genes remained unaltered after pravastatin treatment (Figure 4F). The same results were observed in PM, Fis1, and Mfn2 genes were markedly upregulated by 50-fold while Dnm1l, Ppif, Mfn1, and Opa1 genes remained unaltered after pravastatin treatment (Supplementary Figure S6H). These results indicate pravastatin treatment upregulates genes involved in mitochondrial fission and fusion in both macrophage sources.

### Pravastatin Treatment Impairs Mitochondrial Bioenergetics and Increases Superoxide Anion Production in Macrophages

Pravastatin treatment did not change mitochondrial respiration (Supplementary Figures S7A–F) and glycolytic rates (Supplementary Figures S7G–I) in BMDM. Regarding PM, pravastatin treatment reduced OCR associated with basal respiration (19%), ATP production (19%), and proton leak (19%) (Supplementary Figures S8A–F). No alteration in the glycolytic rates was observed in PM from pravastatin-treated mice (Supplementary Figures S1G–I).

**FIGURE 5:**
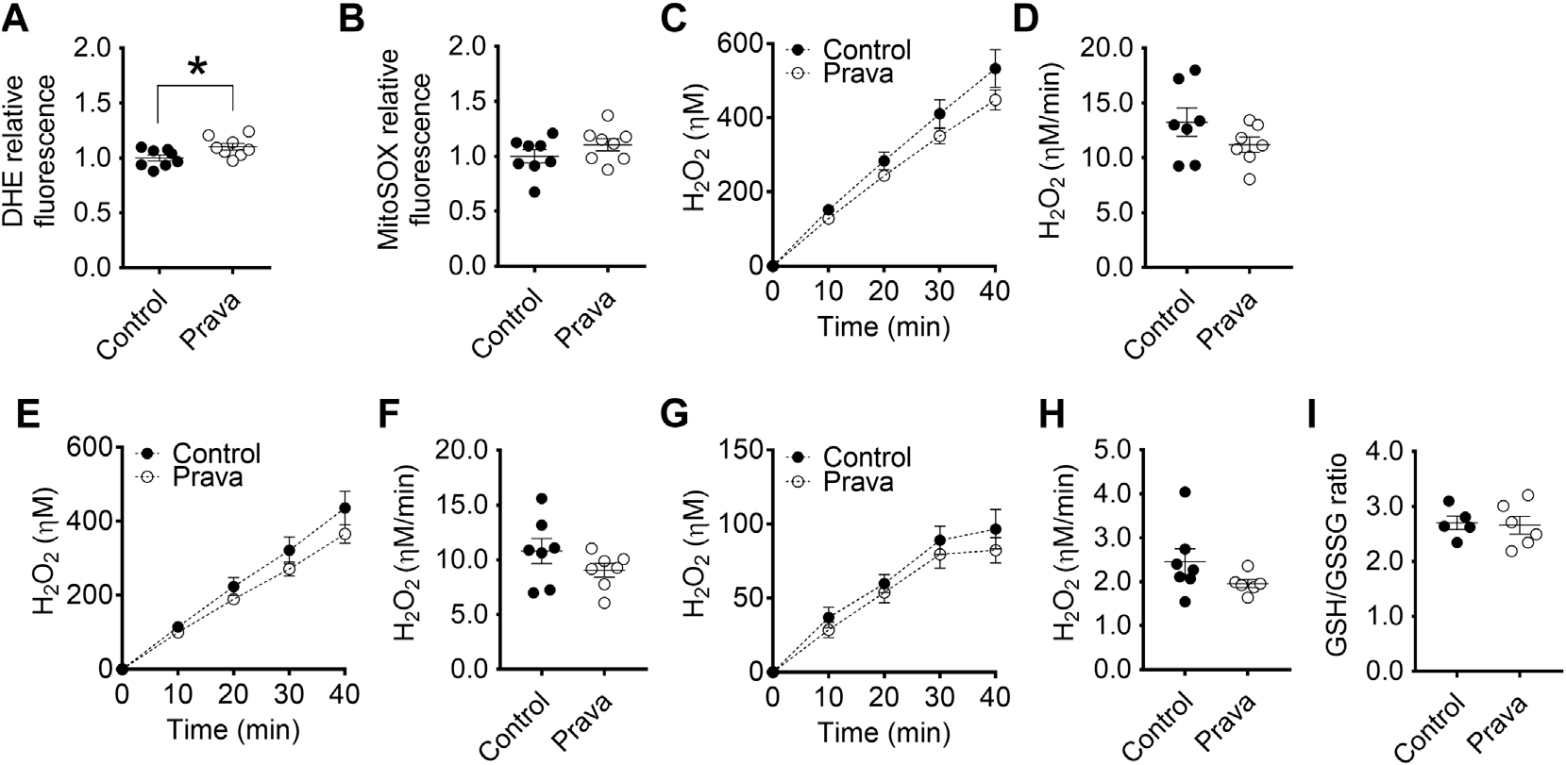
Pravastatin treatment increases global superoxide anion production in BMDM. Detection of global **(A)** and mitochondria-derived **(B)** superoxide anion production in BMDM from non-treated (control) and pravastatin-treated (prava) LDLr^−/−^ mice is shown. Four replicates per mouse were considered, each corresponding to the average of nine fields analyzed by fluorescence microscopy. Control: *n* = 8 mice and prava: *n* = 8 mice. Average curves and rate quantitation of total **(C,D)**, non-mitochondrial **(E,F)**, and mitochondrial release **(G,H)** of hydrogen peroxide (H_2_O_2_). Values were normalized by DNA amount in each well. Three replicates per mouse were considered. Data are expressed as mean ± SE. Control: *n* = 7 mice and prava: *n* = 7 mice. **(I)** Oxidative stress assessed by GSH/GSSG ratio. Control: *n* = 5 mice and prava: *n* = 6 mice. Statistical analyses were performed using two-tailed unpaired Student’s t-test. * represents *p* < 0.05.

Regarding superoxide anion production rates, both BMDM (Figures 5A,B) and PM (Supplementary Figures S9A,B) of pravastatin-treated LDLr^−/−^ mice showed a higher rate of cell (DHE-probed), but not mitochondrial-derived (MitoSOX-probed) superoxide anion production. Pravastatin treatment did not alter the rates of total, non-mitochondrial, and mitochondrial H_2_O_2_ released in both BMDM (Figures 5C–H) and PM (Supplementary Figures S9C–H) when compared to control untreated LDLr^−/−^ mice. No alterations in GSH/GSSG ratio were detected in BMDM (Figure 5I) and PM (Supplementary Figure S9I) of control untreated and pravastatin-treated LDLr^−/−^mice. Thus, in summary, pravastatin treatment decreases mitochondrial respiration in PM and stimulates global superoxide anion production in both PM and BMDM.

### Pravastatin Treatment Decreases Foam Cell Formation and Phagocytosis in Bone Marrow-Derived Macrophages

**FIGURE 6:**
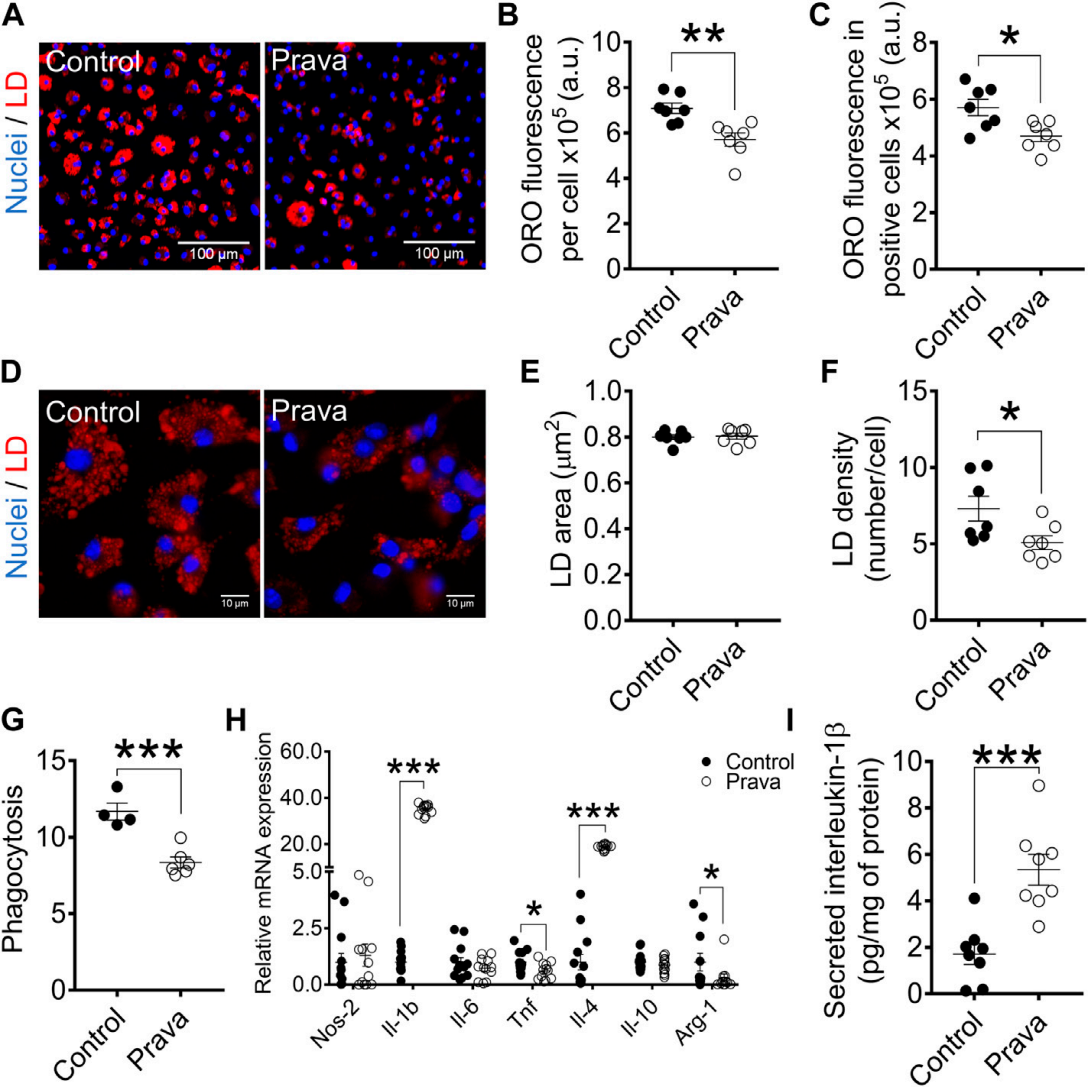
Pravastatin treatment decreases foam cell formation, lipid droplets density, and phagocytosis and modulates either pro- and anti-inflammatory gene expression in BMDM. **(A)** Representative images at 10× magnification of BMDM from non-treated (control) and pravastatin-treated (prava) LDLr^−/−^ mice after incubation with ox-LDL. Neutral lipids were stained with ORO fluorescent dye (red) and nuclei with Hoechst33342 (blue). Quantitative analysis of ORO fluorescence intensity in all cells **(B)** and in positive cells **(C)**. **(D)** Representative images of lipid droplets at 60× magnification and stained with ORO (red) and nuclei with Hoechst33342 (blue). Quantitative analysis of lipid droplets’ size **(E)** and density **(F)** from images displayed in **(D)**. Three replicates per mouse were considered, each corresponding to the average of nine fields analyzed by fluorescence microscopy. Control: *n* = 7 mice and prava: *n* = 7 mice. **(G)** Zymosan phagocytosis. Control: *n* = 4 mice and prava: *n* = 6 mice. Two replicates per mouse. (H) Relative mRNA expression of inflammatory-related genes. Control: *n* = 13 mice and LDLr^−/−^: *n* = 12 mice, with 2 replicates for each gene. Data are expressed as mean ± SE. **(I)** Interleukin-1β (Il-1β) secretion in cell supernatant and normalized by protein content in the cell lysate. Data are expressed as mean ± SE. Control: *n* = 8 mice and prava: *n* = 8 mice, with 2 replicates for each mouse. Statistical analyses were performed using two-tailed unpaired Student’s t-test. *, **, and *** represent *p* < 0.05, 0.01, and 0.001, respectively.

In BMDM, pravastatin treatment reduced in approximately 20% the global ORO fluorescence (Figures 6A,B) and the LD-positive cell ORO fluorescence (Figure 6C). Pravastatin did not change lipid droplets areas (Figures 6D,E), but it decreased by 30% the number of lipid droplets per cell (Figure 6F). Lastly, pravastatin impaired in ∼30% the phagocytic capacity of BMDM (Figure 6G). In PM, pravastatin did not affect global ORO fluorescence (Supplementary Figures S10A,B) and LD-positive cell ORO fluorescence (Supplementary Figure S10C). Lipid droplets size (Supplementary Figures S10D,E) and density (Supplementary Figure S10F) also were not modified by pravastatin in PM. Phagocytosis rates were similar in PM of control and pravastatin-treated LDLr^−/−^ mice (Supplementary Figure S10G).

Together, these results indicate pravastatin treatment reversed the hypercholesterolemia exacerbation of foam cell formation and phagocytosis in BMDM, but not in PM of LDLr^−/−^ mice.

### Pravastatin Treatment Modulates Macrophage Gene Expression of Inflammatory Markers and Increases Secretion of IL-1β

We investigated whether pravastatin treatment could affect macrophage inflammatory gene expression profile in LDLr^−/−^ mice. In fact, pravastatin provoked marked changes in BMDM, leading to a 35-fold increase in Il-1b, 39% decrease in Tnf, 19-fold increase in Il-4, and 87% decrease in Arg-1 levels. Pravastatin did not change NO synthase-2 (Nos-2), Il-6, and Il-10 levels in BMDM (Figure 6H). Pravastatin treatment also induced a 2-fold increase in the interleukin-1β (Il-1β) secretion by BMDM of LDLr^−/−^ mice (Figure 6I). In PM, pravastatin enhanced the gene expression of either pro- or anti-inflammatory markers, including a 5.6-fold increase in Nos-2, 6.3-fold increase in Il-1b, 2.3-fold increase in Il-6, and 12.7-fold increase in Il-4 levels. Pravastatin treatment did not affect Tnf, Il-10, and Arg-1 expression in PM (Supplementary Figure S10H). Pravastatin treatment also induced a 5.5-fold increase in Il-1β secretion by PM of LDLr^−/−^ mice (Supplementary Figure S10I). These results show that pravastatin treatment affects the PM and BMDM inflammatory gene pattern of expression and increases secretion of Il-1β, a classical pro-inflammatory behavior.

## Discussion

ER–mitochondria communication plays a role in immune signaling (Zhou et al., 2011; Subramanian et al., 2013). In this work, we verified that hypercholesterolemia increases and pravastatin treatment decreases the number of contact sites at the MAM in both types of macrophages, PM and BMDM. ER pumps calcium towards mitochondria at MAM micro-domains, avoiding cytoplasmic chelators sequestrate calcium. Mitochondrial calcium regulates tricarboxylic acid cycle (TCA) enzymes’ activity, including pyruvate dehydrogenase kinase, isocitrate dehydrogenase, and α-ketoglutarate dehydrogenase (Denton, 2009). Conversely, mitochondria calcium overload leads to permeability transition and cell death (Vercesi et al., 2018; Oliveira and Vercesi, 2020). Thus, MAM contact sites number and calcium pumping into mitochondria may be coordinately regulated supporting organelles’ proper functioning (Lopez-Crisosto et al., 2017). Based on that, we speculate that hypercholesterolemia might affect calcium influx toward mitochondria in macrophages.

Diseases characterized by local or systemic inflammation may cause MAM abnormalities in certain tissues (Missiroli et al., 2018). This is the case of peripheral blood CD14^+^ mononuclear cells of subjects carrying rheumatoid arthritis and coronary artery disease, which display an increased number of MAM contact sites when compared to healthy individuals (Zeisbrich et al., 2018). In this work, we verified that hypercholesterolemia increases the number of contact sites between the ER and mitochondria in BMDM. Considering that changes in MAM stability persisted after 7 days of stem cell differentiation into macrophages *in vitro*, this likely reflects epigenetic mechanisms. Our results agree with earlier findings showing hypercholesterolemia triggers trained immunity in hematopoietic cells via activation of Nlrp3-inflammasome (Duewell et al., 2010; Bekkering et al., 2014; Christ et al., 2018). Trained immunity depends on the mevalonate, and thus, pravastatin prevents the induction of trained immunity memory by inhibiting the 3-hydroxy-3-methyl-glutaryl-coenzyme A (HMG-CoA) conversion into mevalonate (Bekkering et al., 2018). Here, we showed that *in vivo* pravastatin treatment restored the number of ER–mitochondria contact sites in BMDM to a value similar to that found in macrophages of WT mice. Thereby, we suggest enhanced MAM contact sites as a new signature of trained immunity caused by hypercholesterolemia in stem cell-derived macrophages.

Mitochondria fusion and fission are dynamic processes assisted by the ER (Friedman et al., 2011; Guo et al., 2018). In PM, hypercholesterolemia did not affect the interaction between the ER and mitochondria, but it increased organelle branching degree. Pravastatin treatment decreased the number of contact sites between the ER and mitochondria in PM of LDLr^−/−^ mice and had no significant effect on mitochondria length or branching. In BMDM, hypercholesterolemia increased ER–mitochondria interaction and did not affect mitochondria length or branching. Pravastatin-treated LDLr^−/−^ mice reduced inter-organelle interactions and enhanced the mitochondrial branching degree in BMDM. Based on that, we suggest that changes in ER–mitochondria interaction are associated with changes in mitochondria network morphology in BMDM, but not in PM. These distinct responses might be related to each differentiation process of PM (*in vivo*) and BMDM (*in vitro*).

Pravastatin treatment, but not hypercholesterolemia, upregulated Mfn2 and Fis1 gene expression in both macrophages of LDLr^−/−^ mice. Four concurrent scenarios might explain this condition. 1) Mfn2 and Fis1 upregulation suggests mitochondria fuse and fragment at similar rates; 2) Mfn2 gene is abundantly expressed in macrophages (Tur et al., 2020), and despite its function in fusing neighboring mitochondria (Santel and Fuller, 2001), Mfn2 dimers also play a role in MAM formation by tethering ER membranes to mitochondria (de Brito and Scorrano, 2008). Considering both PM and BMDM from pravastatin-treated mice presented reduced ER–mitochondria interaction in association with Mfn2 gene upregulation, it is likely that Mfn2 gene increase might compensate for the loss in Ip3r1-Vdac1 interaction; 3) another possibility involves the inhibition of Mfn2 by Fis1 (Yu et al., 2019) to counteract Mfn2 high levels and keep mitochondria morphology unaltered; and 4) last but not the least, Mfn2 and Fis1 prenylation is mandatory to anchor these proteins on the mitochondria surface where they orchestrate mitochondrial dynamics. However, pravastatin blocks the mevalonate pathway and reduces isoprenoid synthesis, i.e., prenylation substrates (Laufs et al., 2002; Wang and Casey, 2016). Thus, Mfn2 and Fis1 upregulation suggest dysfunctional proteins might accumulate in the cytosol. The gene expression of other proteins prone to prenylation, such as Mfn1, Opa1 and Drp1 (Schrepfer and Scorrano, 2016), were not affected by pravastatin.

In BMDM, hypercholesterolemia increased five times the ER–mitochondria interaction and macrophage’s capacity to take up ox-LDL. Conversely, pravastatin treatment of LDLr^−/−^ mice reduced the number of ER–mitochondria contact sites and ox-LDL intake. The link between MAM stability and foam cell formation may involve the acyl-CoA:cholesterol acyltransferase 1 (Acat1). Acat1 converts free cholesterol into cholesteryl ester and shifts the equilibrium towards lipid droplets’ formation. This allows macrophages to store cholesterol in a less cytotoxic way (Puglielli et al., 2001). Acat1 distributes along with MAM in certain tissues (Rusiñol et al., 1994). Acat1 activity maximizes as ER–mitochondria interaction increases and reduces as organelles move away (Area-Gomez et al., 2012). Based on that, we suggest hypercholesterolemia and pravastatin treatment modulates MAM stability, which affects Acat1 activity and foam cell formation.

Hypercholesterolemia reduced superoxide anion production in PM, but increased hydrogen peroxide release in both PM and BMDM. The sources of hydrogen peroxide were extra-mitochondrial in PM and mitochondrial in BMDM. These findings are in line with earlier studies showing increased oxidant production in several tissues of LDL^−/−^ mice, including linfomononuclear cells (Oliveira et al., 2005; Paim et al., 2008). Chronic *in vivo* pravastatin enhanced global cell (DHE), but not the mitochondria-derived (MitoSOX) superoxide anion generation in both PM and BMDM. However, pravastatin did not change the hydrogen peroxide release that was already elevated in macrophages of LDLr^−/−^ mice before pravastatin treatment. Thus, it is likely these cells are under oxidative stress in both conditions, treated and non-treated with pravastatin, as proposed recently (Oliveira and Vercesi, 2020). Accordingly, pravastatin-treated LDLr^−/−^ mice present oxidative stress in the muscle (Busanello et al., 2017), liver (Marques et al., 2018), and pancreatic islet (Lorza-Gil et al., 2016; Lorza-Gil et al., 2019).

Statins inhibit mitochondrial respiration in different tissues (Mullen et al., 2011; Kwak et al., 2012; la Guardia et al., 2013; Schirris et al., 2015; Fišar et al., 2016; Busanello et al., 2017). However, statin effects on mitochondria respiration in macrophages has been overlooked. We show here that, while hypercholesterolemia did not affect mitochondrial respiration, pravastatin treatment reduced mitochondrial respiration rates in primary PM, but not in BMDM. These results suggest that pravastatin does not affect BMDM mitochondrial respiration or its effects disappear (or are compensated) during the 7 days of differentiation. The L929 conditioned medium directs bone marrow differentiation towards an anti-inflammatory polarized phenotype. Thus, it is possible that pravastatin effect may depend on the macrophage activation state. Kim et al. (Kim et al., 2006) reported that lipopolysaccharide-activated RAW246.7 macrophages are sensitive to low doses of simvastatin, present reduced mitochondrial membrane potential, and increased mitochondrial permeability transition in a calcium-dependent way (Kowaltowski et al., 2001; Velho et al., 2006). Thus, pro-inflammatory macrophages, although more glycolytic, seem to be more sensitive to statins than anti-inflammatory macrophages.

Hypercholesterolemia and pravastatin treatment modulated the expression of both pro- and anti-inflammatory related genes, in a distinct way, in both BMDM and PM, making a phenotype interpretation quite difficult. Noteworthy, pravastatin *in vivo* treatment markedly increased interleukin-1β gene expression and secretion in both macrophage sources of LDLr^−/−^ mice. Since pravastatin did not ameliorate and actually worsened hypercholesterolemia-induced oxidative stress, this may be causally related to the increased Il-1β in both BMDM and PM. It is generally accepted that generation of oxidants, either by inhibition of mitochondrial respiration or by activation of phagosomal NADPH oxidase, regulates NF-κB activity and induces inflammatory cytokine gene expression, including Il-1β (Park et al., 2004; Kim et al., 2008). In addition, Tassi et al. (2009) have shown that IL-1β processing and secretion is regulated by a biphasic redox event including a prompt oxidative stress and a delayed antioxidant response. Another possible explanation is related to the statins’ effect of reducing geranylgeranyl-pyrophosphate availability. GGTase-I enzyme uses geranylgeranyl-pyrophosphate to modify Rac1. Prenylated Rac1 impairs innate immune response, while non-prenylated Rac1 is hyperactive and drives Il-1β secretion in differentiated macrophages. Thus, pravastatin has similar effects to GGTase-I ablation in macrophages (Lindholm and Nilsson, 2007; Akula et al., 2019; Fu et al., 2019; Healy et al., 2020). Therefore, although pravastatin decreased foam cell formation, the unexpected increase of IL-1β secretion could, in fact, be detrimental for other atherosclerosis-relevant cell types, such as endothelial cells.

The main findings in the present study show hypercholesterolemia increased the number of MAM contact sites, foam cell formation, and phagocytosis, whereas pravastatin reversed all these parameters in BMDM. However, in PM, hypercholesterolemia did not affect the number of MAM contact sites, foam cell formation, and phagocytic activity. Although pravastatin reduced the number of MAM contact sites in PM, it had no significant effect on foam cell formation or phagocytic activity in PM. In addition, pravastatin increased mitochondrial branching in BMDM and increased markedly the expression of the mitochondrial dynamics-related genes Mfn2 and Fis1 in both macrophages. The differences of results coming from the two cell models are likely related to the heterogeneity and plasticity of these cells, which are highly specialized in sensing the microenvironment and modify their properties accordingly. Thus, it is not surprising and rather expected to find some different responses between BMDM and PM. In addition, one plausible reason may be related to the differentiation process. While PM is a final *in vivo* differentiated primary cell and, thus, life-long exposed to the hypercholesterolemic milieu, BMDM differentiated *in vitro*, in the absence of hypercholesterolemic media, and, thus, reflects epigenetic alterations induced by hypercholesterolemia during the *in vivo* precursor stage of the cells. Keeping this in mind, the nature of differentiation, whether *in vivo* or *in vitro*, is likely to affect the final macrophage phenotype. It is also important to show that not all sources of macrophage (bone marrow, blood monocytes, spleen mononuclear, and other tissue-resident macrophages) respond the same way to hypercholesterolemia and pravastatin treatment.

In summary, our results show that hypercholesterolemia and pravastatin treatment affect macrophage mitochondria structure and function, as well as their interaction with the ER. These effects impact on macrophage conversion rates to foam cell and macrophage phagocytic capacity. To our knowledge, this is the first evidence supporting that both hypercholesterolemia and statin treatment modulate MAM stability and mitochondrial network morphology in macrophages. Pravastatin promoted changes towards the control profile rescuing mitochondria–ER interactions. These findings associate MAM changes with known mechanisms involved in atherosclerosis progression and resolution.

## Limitations of This Study

Hypercholesterolemia and pravastatin changed the interaction pattern between Ip3r1 and Vdac1 in macrophages. Ip3r1-Vdac1 is a well-characterized tethering complex involved in ER–mitochondria interaction. For this reason, many studies use their proximity as indicative of MAM stability itself. However, we cannot warrant that hypercholesterolemia/pravastatin changes overall MAM extension because we did not test other tethering complexes, such as mitofusins. In addition, the LDLr^−/−^ mice model used in this study is hypercholesterolemic since embryo formation. Thus, we do not know whether pravastatin affects MAM stability because it reduces plasma cholesterol levels or because it blocks trained immunity acquisition in macrophages. Another relevant point regards mice genetic background. C57BL/6J wild-type and LDLr^−/−^ (B6.129S7-Ldlr < tm1Her./J) mice lines do not share the exact same genetic background. Thus, we may not exclude potential genetic interferences in some of the results when WT is compared to LDLr^−/−^. However, C57BL/6J is indicated by the Jackson Laboratory as the closest available control for B6.129S7-Ldlr < tm1Her./J mice.

## Data Availability

The original contributions presented in the study are included in the article/Supplementary Material, further inquiries can be directed to the corresponding author.
