# Supplementary material for: In Vivo Pravastatin Treatment Reverses Hypercholesterolemia Induced Mitochondria-Associated Membranes Contact Sites, Foam Cell Formation, and Phagocytosis in Macrophages
Source: Front Mol Biosci. 2022 Mar 15;9:839428. doi: 10.3389/fmolb.2022.839428 (PMC8965079; doi:10.3389/fmolb.2022.839428)
Supplement: Supplementary file 1 [file DataSheet1.pdf]

Supplementary Material

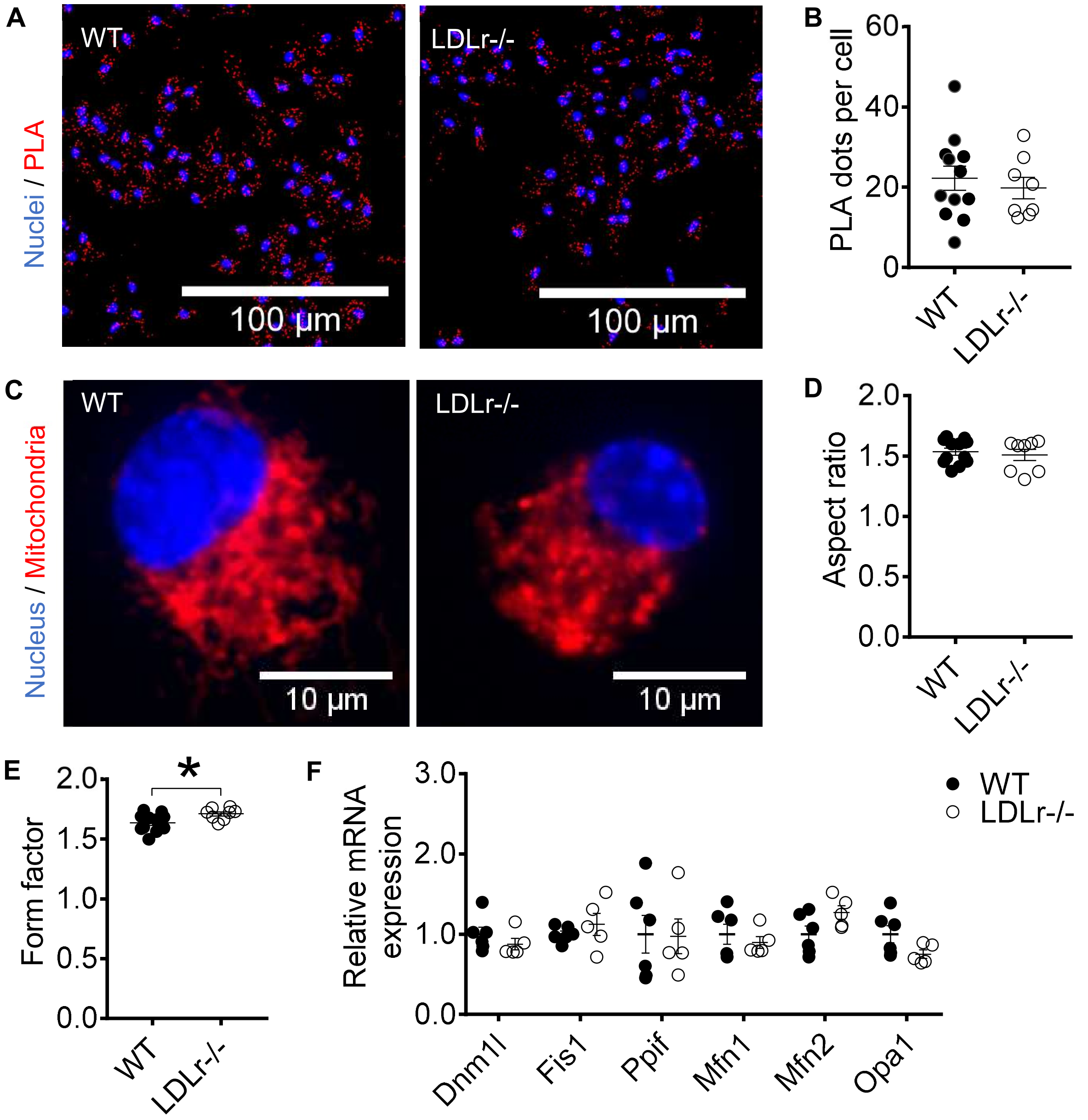

Supplementary Figure S1. Hypercholesterolemia increases mitochondrial branching in PM. **(A)** Representative proximity ligation assay (PLA) images at 40x magnification and **(B)** quantitative analysis of Ip3r1-Vdac1 interactions in PM from WT and LDLr-/- mice. Ip3r1-Vdac1 interactions detected through PLA assay were labeled with Cyanine 5 (red) and nuclei with DAPI (blue). Two replicates per mouse, each corresponding to the average of nine fields analyzed by fluorescence microscopy. WT (n=12 mice) and LDLr-/- (n=8 mice). **(C)** Representative images of mitochondria network at 60x magnification and quantitative analysis of mitochondria aspect ratio **(D)** and form factor **(E)**. Four replicates per mouse, each corresponding to the average of 9 fields analyzed by fluorescence microscopy. Mitochondria stained with MitoTracker (red) and nuclei with Hoechst 33342 (blue). WT (n=12 mice) and LDLr-/- (n=8 mice). **(F)** Relative gene expression of mitochondrial fusion and fission markers. WT (n=6 mice) and LDLr-/- (n=5 mice). Data are expressed as Mean ± SE. Statistical analyses were performed using a two-tailed unpaired Student-t test. * with $p<0.05$.

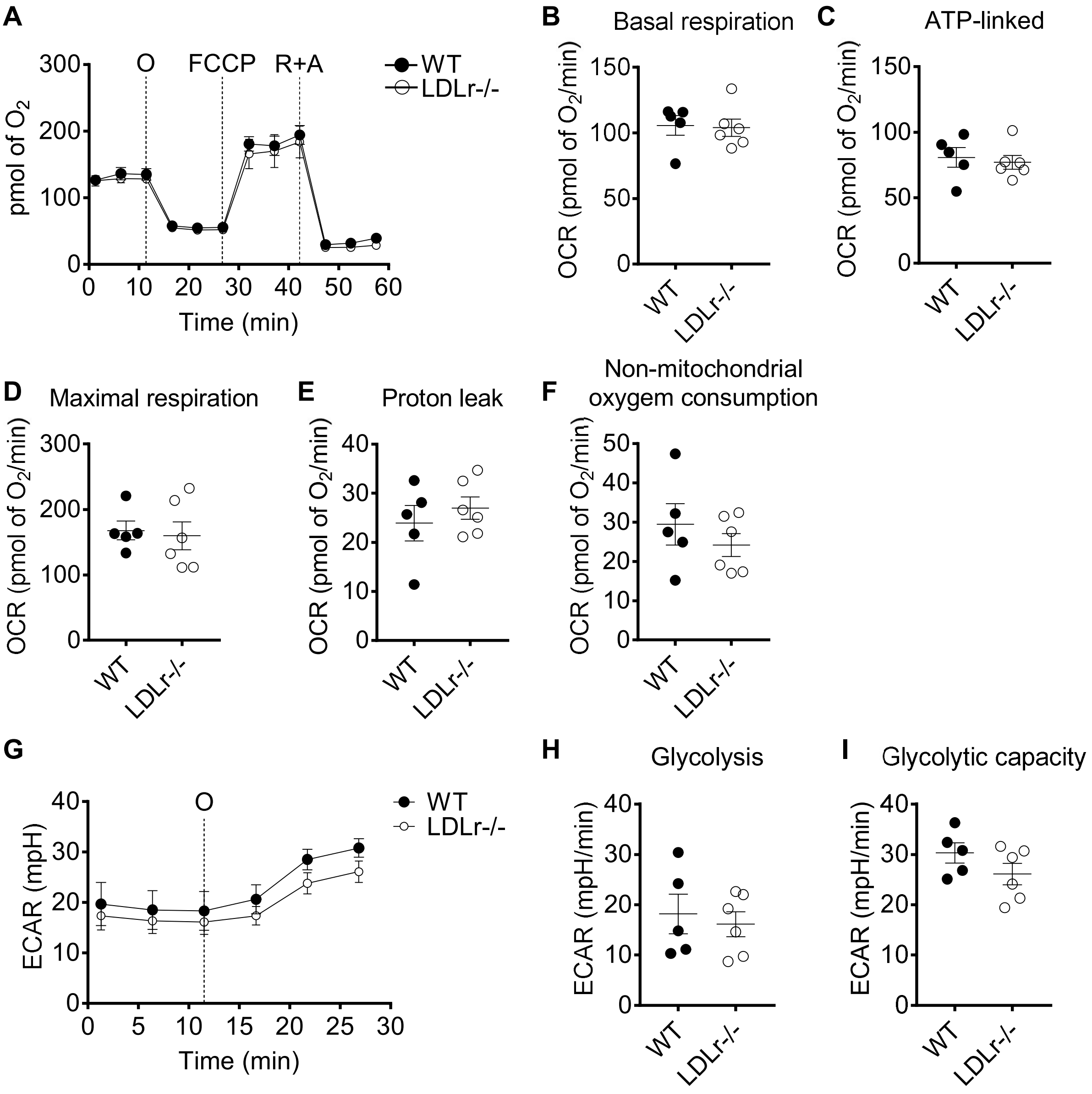

Supplementary Figure S2. Hypercholesterolemia does not affect mitochondrial respiration and glycolytic function in BMDM. (A) Average curves of oxygen consumption rates (OCR) of BMDM from WT and LDLr-/- mice. Oligomycin (O), FCCP, rotenone plus antimycin-A (R+A) were sequentially injected to assess mitochondrial respiratory rates associated to specific states: **(B)** basal respiration, **(C)** ATP production, **(D)** maximal respiration, **(E)** proton leak and **(F)** non-mitochondrial oxygen consumption. **(G)** Average curves of extracellular acidification rate (ECAR) of BMDM from WT and LDLr-/- mice. **(H)** Glycolysis and **(I)** glycolytic capacity. OCR and ECAR values were normalized by the respective DNA amount in each well. Three replicates per mouse. Data are expressed as Mean ± SE. WT (n=5 mice) and LDLr-/- (n=6 mice) for OCR and ECAR assays. Statistical analyses were performed using a two-tailed unpaired Student-t test.

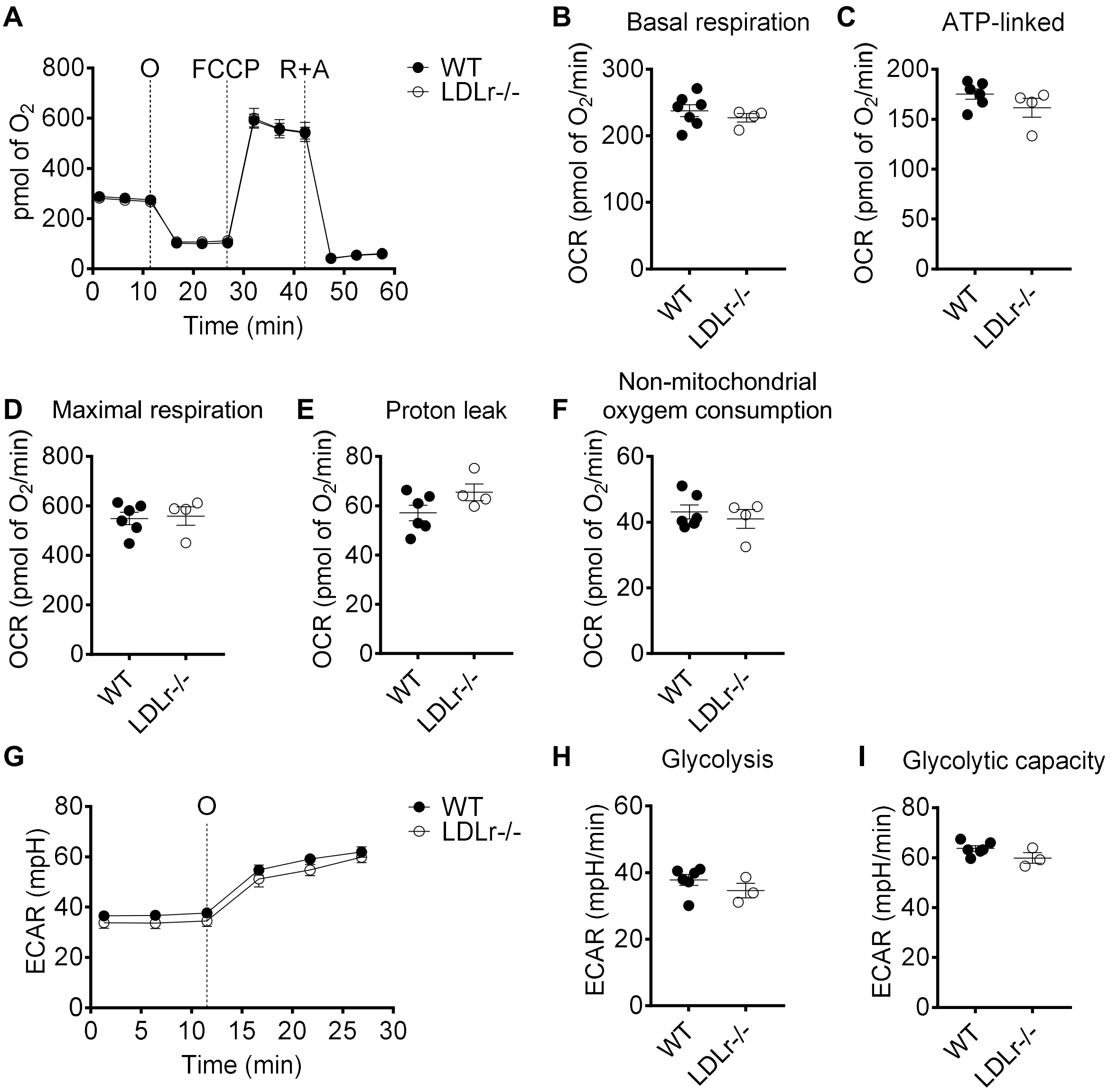

Supplementary Figure S3. Hypercholesterolemia does not affect mitochondrial respiration and glycolytic function in PM. (A) Average curves of oxygen consumption rates (OCR) of PM from WT and LDLr-/- mice. Oligomycin (O), FCCP, rotenone plus antimycin-A (R+A) were sequentially injected to assess mitochondrial respiratory rates associated to specific states: **(B)** basal respiration, **(C)** ATP production, **(D)** maximal respiration, **(E)** proton leak and **(F)** non-mitochondrial oxygen consumption. WT (n=6 mice) and LDLr-/- (n=4 mice). **(G)** Average curves of extracellular acidification rate (ECAR) of PM from WT and LDLr-/- mice. **(H)** Glycolysis and **(I)** glycolytic capacity. WT (n=6 mice) and LDLr-/- (n=3 mice). OCR and ECAR values were normalized by the respective DNA amount in each well. Three replicates per mouse. Data are expressed as Mean ± SE. Statistical analyses were performed using a two-tailed unpaired Student-t test.

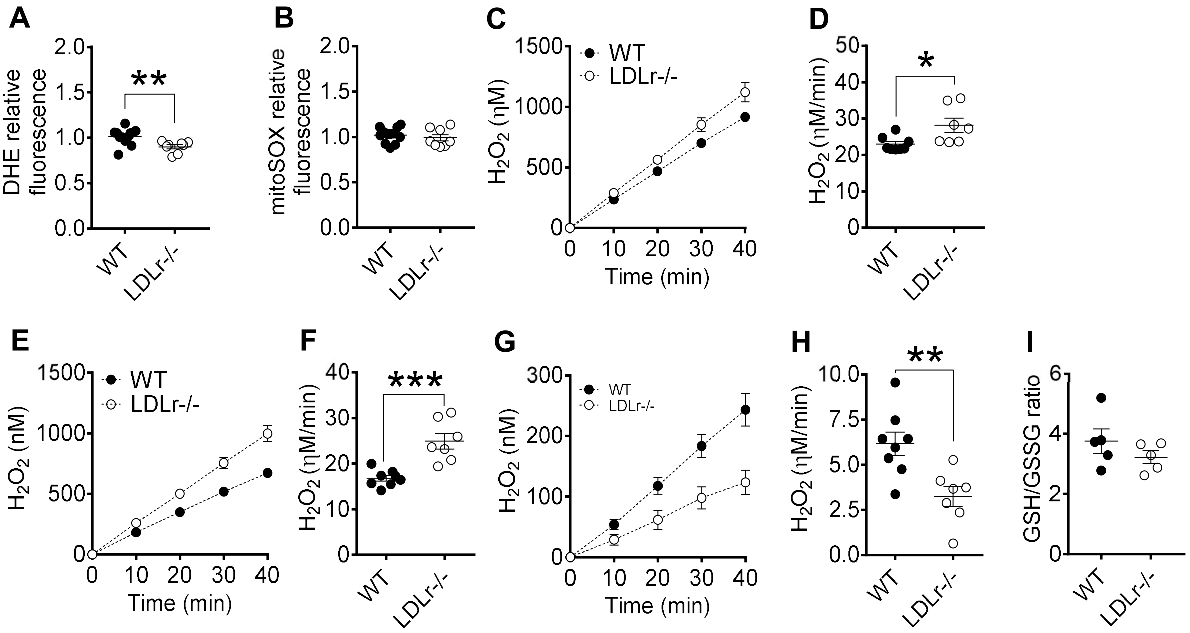

Supplementary Figure S4. Hypercholesterolemia reduces global superoxide anion production and modulates hydrogen peroxide release in PM. Detection of global **(A)** and mitochondria-derived **(B)** superoxide anion production in PM from WT and LDLr-/- mice. Four replicates per mouse, each corresponding to the average of 9 fields analyzed by fluorescence microscopy. WT (n=12 mice) and LDLr-/- (n=8 mice). Average curves and rate quantitation of total **(C,D)**, non-mitochondrial **(E,F)** and mitochondrial **(G,H)** release of hydrogen peroxide (H_2O_2). Values were normalized by DNA amount in each well. Three replicates per mouse. Data are expressed as Mean ± SE. WT (n=8 mice) and LDLr-/- (n=7 mice). **(I)** Oxidative stress assessed by GSH/GSSG ratio. WT (n=5 mice) and LDLr-/- (n= 5 mice). Statistical analyses were performed using a two-tailed unpaired Student-t test. *, **, *** with $p<0.05$, 0.01 and 0.01, respectively.

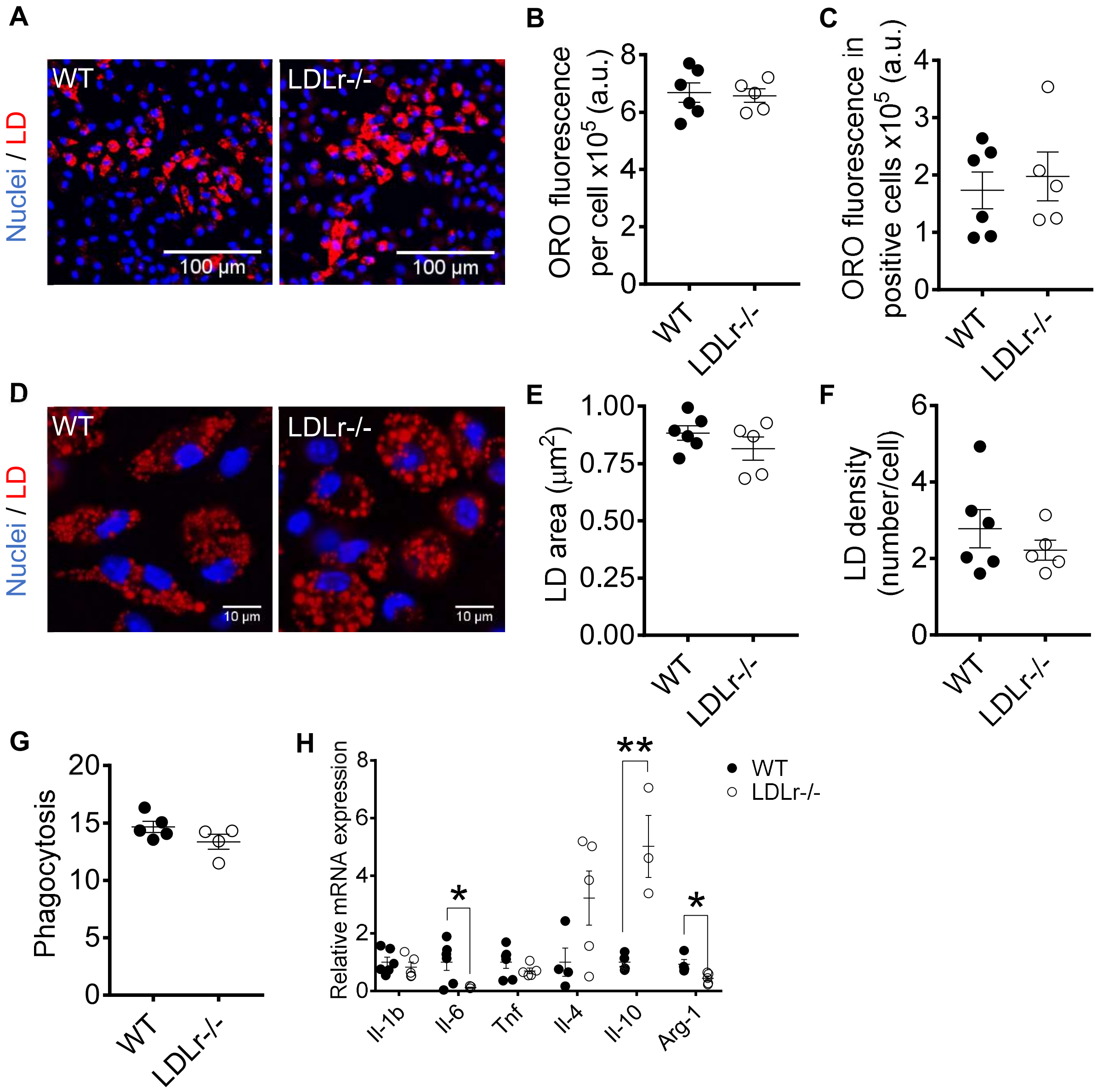

Supplementary Figure S5. Hypercholesterolemia modulates pro- and anti-inflammatory gene expression in PM. (A) Representative images at 10x magnification of PM from WT and LDLr-/- mice after incubation with ox-LDL. Neutral lipids were stained with ORO fluorescent dye (red) and nuclei with Hoechst33342 (blue). Quantitative analysis of ORO fluorescence intensity in all cells **(B)** and in LD positive cells **(C)**. **(D)** Representative images of lipid droplets at 60x magnification and stained with ORO (red) and nuclei with Hoechst33342 (blue). Quantitative analysis of lipid droplets' size **(E)** and density **(F)** from images displayed in the panel D. Three replicates per mouse, each corresponding to the average of nine fields analyzed by fluorescence microscopy. WT (n=6 mice) and LDLr-/- (n=5 mice). **(G)** Zymosan phagocytosis. WT (n=5 mice) and LDLr-/- (n=4 mice). Two replicates per mouse. **(H)** Relative mRNA expression of inflammatory related genes. WT (n=6 mice) and LDLr-/- (n=5 mice), with 2 replicates for each gene. Data are expressed as Mean ± SE. Statistical analyses were performed using two-tailed unpaired Student-t-test. *, ** with $p<0.05$ and 0.01, respectively.

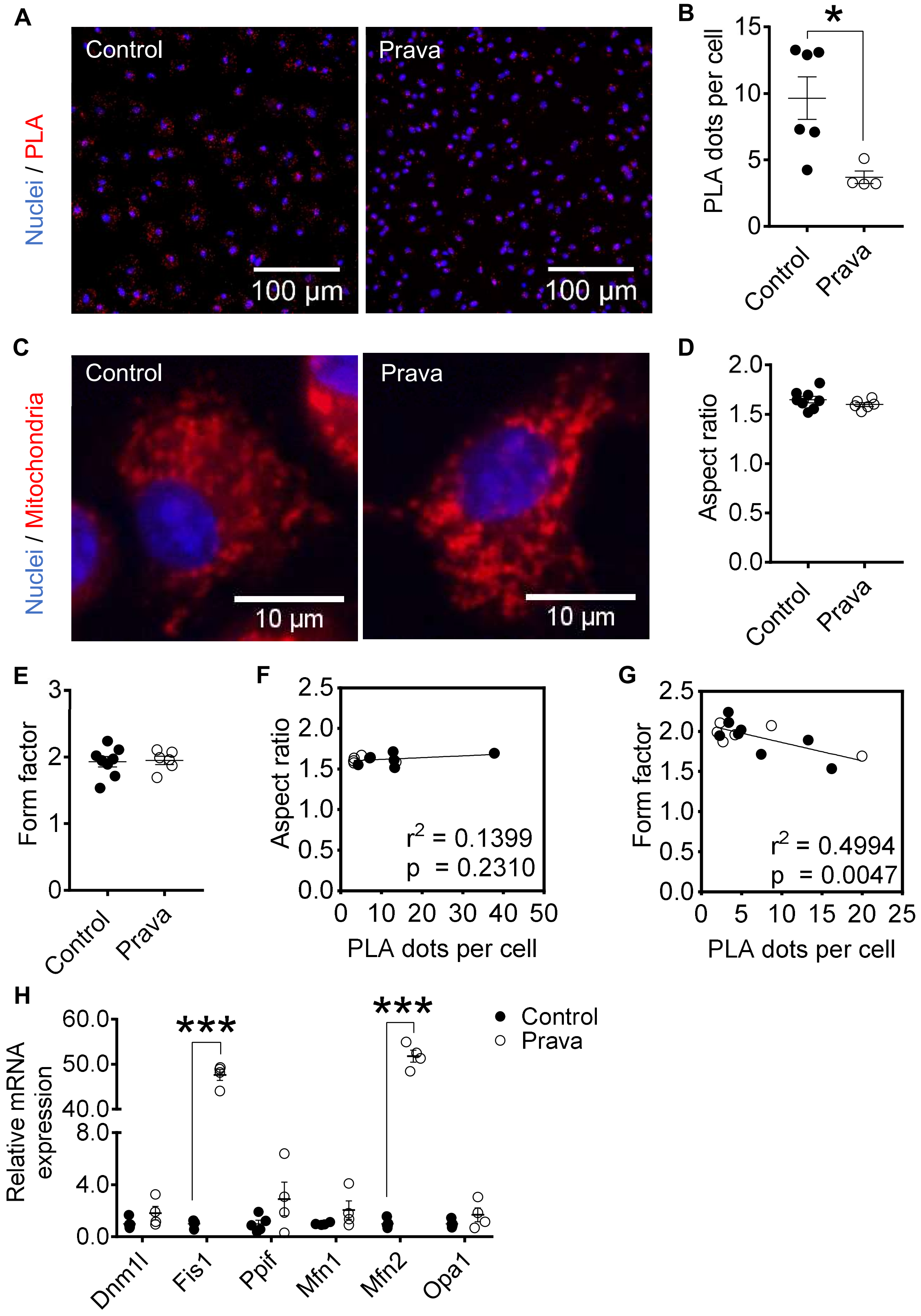

Supplementary Figure S6. Pravastatin treatment decreases ER-mitochondria interaction and upregulates Fis1 and Mfn2 gene expression in PM. (A) Representative proximity ligation assay (PLA) images at 40x magnification and **(B)** quantitative analysis of Ip3r1-Vdac1 interactions in PM from non-treated (Control) and pravastatin-treated (Prava) LDLr-/- mice. Ip3r1-Vdac1 interactions detected through PLA assay were labeled with Cyanine 5 (red) and nuclei with DAPI (blue). Two replicates per mouse, each corresponding to the average of nine fields analyzed by fluorescence microscopy. Control (n=6 mice) and Prava (n=4 mice).

(C) Representative images of mitochondria network at 60x magnification and quantitative analysis of mitochondria aspect ratio **(D)** and form factor **(E)**. Four replicates per mouse, each corresponding to the average of 9 fields analyzed by fluorescence microscopy. Mitochondria stained with MitoTracker (red) and nuclei with Hoechst 33342 (blue). Control (n=8 mice) and Prava (n=6 mice). Statistical analyses were performed using a two-tailed unpaired Student-t test. * $p<0.05$. Correlation analyses between Ip3r1-Vdac1 interactions detected by PLA and mitochondria aspect ratio **(F)** and form factor **(G)**. Control (empty circles) and Prava (filled circles). Statistical analyses using Person's correlation test. **(H)** Relative gene expression of mitochondrial fusion and fission markers. Control (n=5 mice) and Prava (n=4 mice). Data are expressed as Mean ± SE. Statistical analyses were performed using a two-tailed unpaired Student-t test. *, *** with $p<0.05$ and 0.001, respectively.

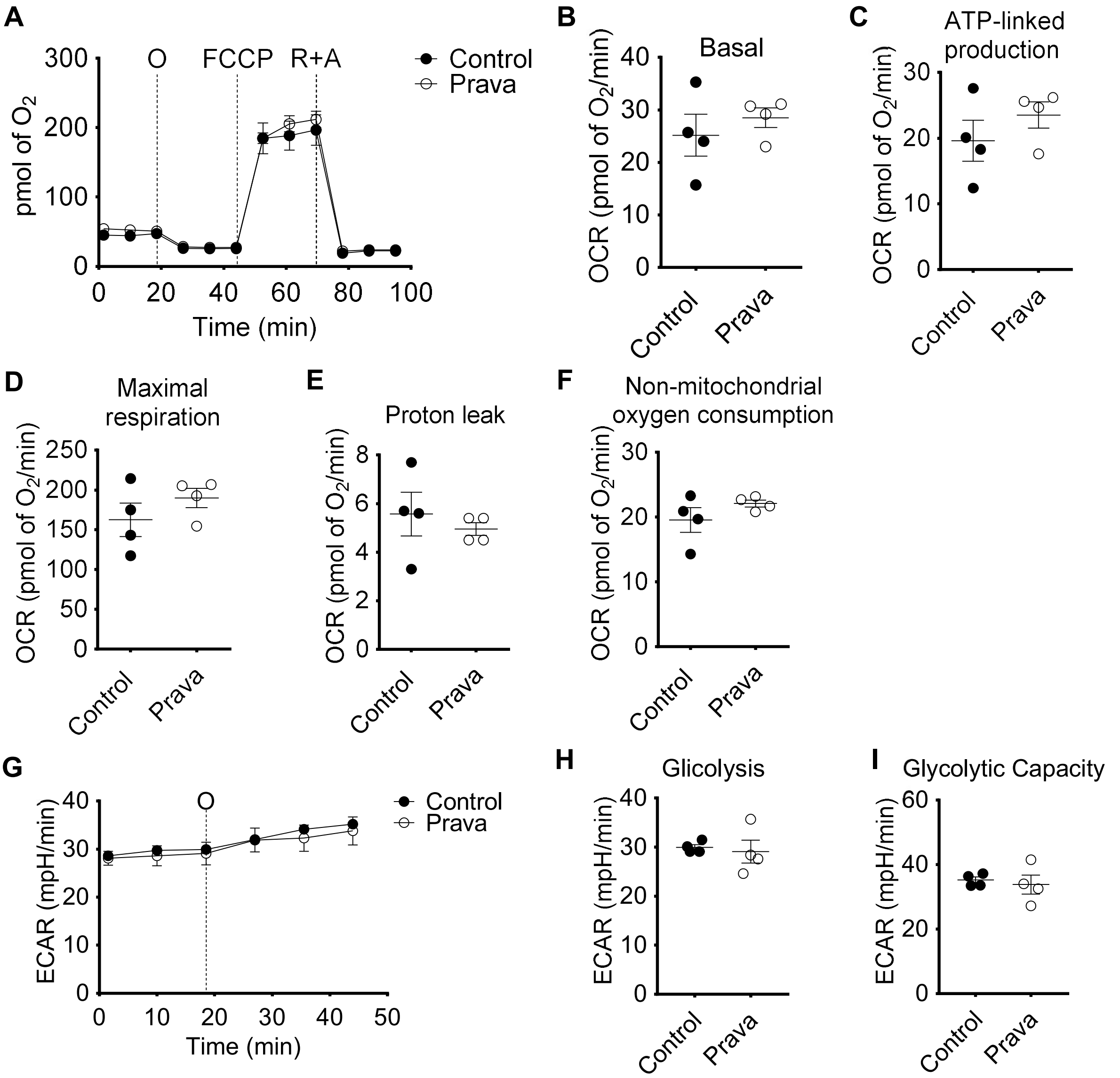

Supplementary Figure S7. Pravastatin treatment does not affect mitochondrial respiration and glycolytic function in BMDM. (A) Average curves of oxygen consumption rates (OCR) of BMDM from non-treated (Control) and pravastatin-treated (Prava) LDLr-/- mice. Oligomycin (O), FCCP, rotenone plus antimycin-A (R+A) were sequentially injected to assess mitochondrial respiratory rates associated to specific states: **(B)** basal respiration, **(C)** ATP production, **(D)** maximal respiration, **(E)** proton leak and **(F)** non-mitochondrial oxygen consumption. **(G)** Average curves of extracellular acidification rate (ECAR) of BMDM from Control and Prava mice. **(H)** Glycolysis and **(I)** glycolytic capacity. OCR and ECAR values were normalized by the respective DNA amount in each well. Three replicates per mouse. Data are expressed as Mean ± SE. Control (n=4 mice) and Prava (n=4 mice) for OCR and ECAR assays. Statistical analyses were performed using a two-tailed unpaired Student-t test.

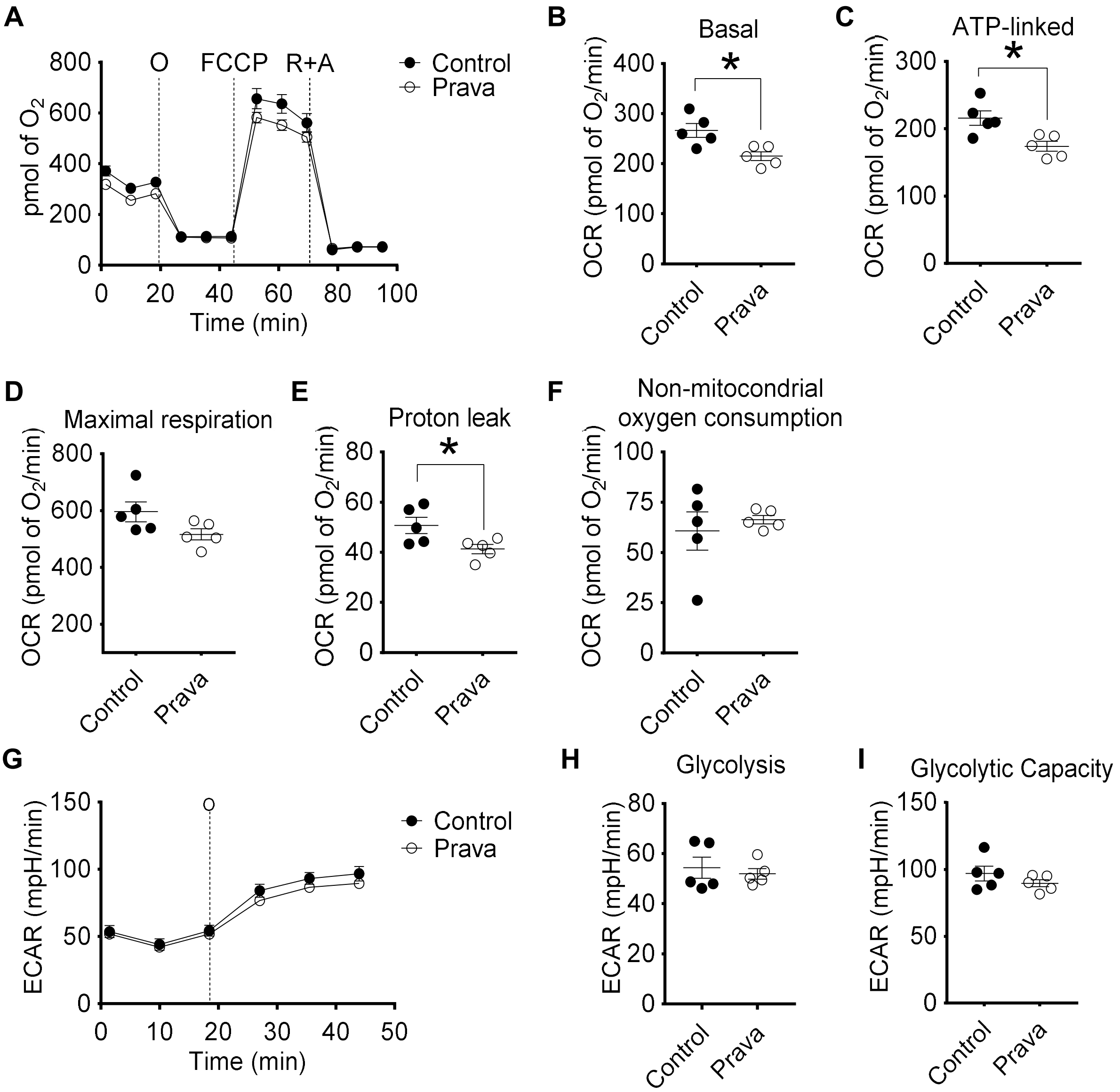

Supplementary Figure S8. Pravastatin treatment reduces mitochondrial respiration in PM. **(A)** Average curves of oxygen consumption rates (OCR) of PM from non-treated (Control) and pravastatin-treated (Prava) LDLr-/- mice. Oligomycin (O), FCCP, rotenone plus antimycin-A (R+A) were sequentially injected to assess mitochondrial respiratory rates associated to specific states: **(B)** basal respiration, **(C)** ATP production, **(D)** maximal respiration, **(E)** proton leak and **(F)** non-mitochondrial oxygen consumption. **(G)** Average curves of extracellular acidification rate (ECAR) of PM from Control and Prava mice. **(H)** Glycolysis and **(I)** glycolytic capacity. OCR and ECAR values were normalized by the respective DNA amount in each well.
Three replicates per mouse. Data are expressed as Mean ± SE. Control (n=5 mice) and Prava (n=5 mice) for OCR and ECAR assays. Statistical analyses were performed using a two-tailed unpaired Student-t test. * with $p<0.05$.

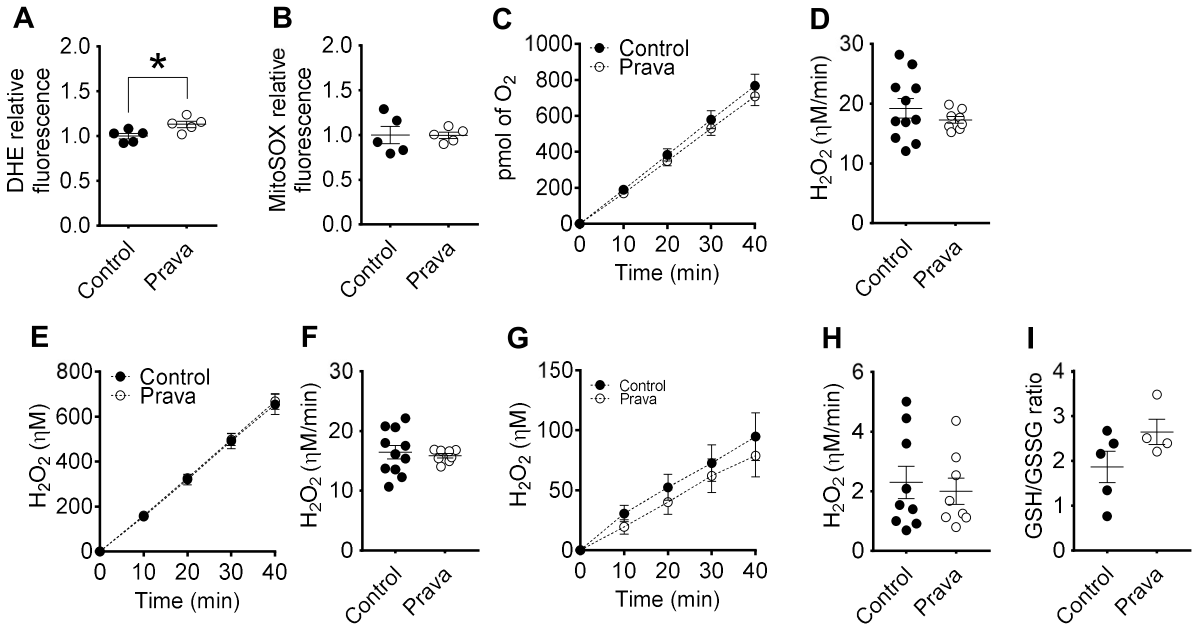

Supplementary Figure S9. Pravastatin treatment increases global superoxide anion production in PM. Detection of global **(A)** and mitochondria-derived **(B)** superoxide anion production in PM from non-treated (Control) and pravastatin-treated (Prava) LDLr-/- mice. Four replicates per mouse, each corresponding to the average of 9 fields analyzed by fluorescence microscopy. Control (n=5 mice) and Prava (n=5 mice). Average curves and rate quantitation of total **(C,D)**, non-mitochondrial **(E,F)** and mitochondrial **(G,H)** release of hydrogen peroxide (H_2O_2). Values were normalized by DNA amount in each well. Three replicates per mouse. Data are expressed as Mean ± SE. Control (n=11 mice) and Prava (n=8 mice). **(I)** Oxidative stress assessed by GSH/GSSG ratio. Control (n=5 mice) and Prava (n=4 mice). Statistical analyses were performed using a two-tailed unpaired Student-t test. * with $p<0.05$ respectively.

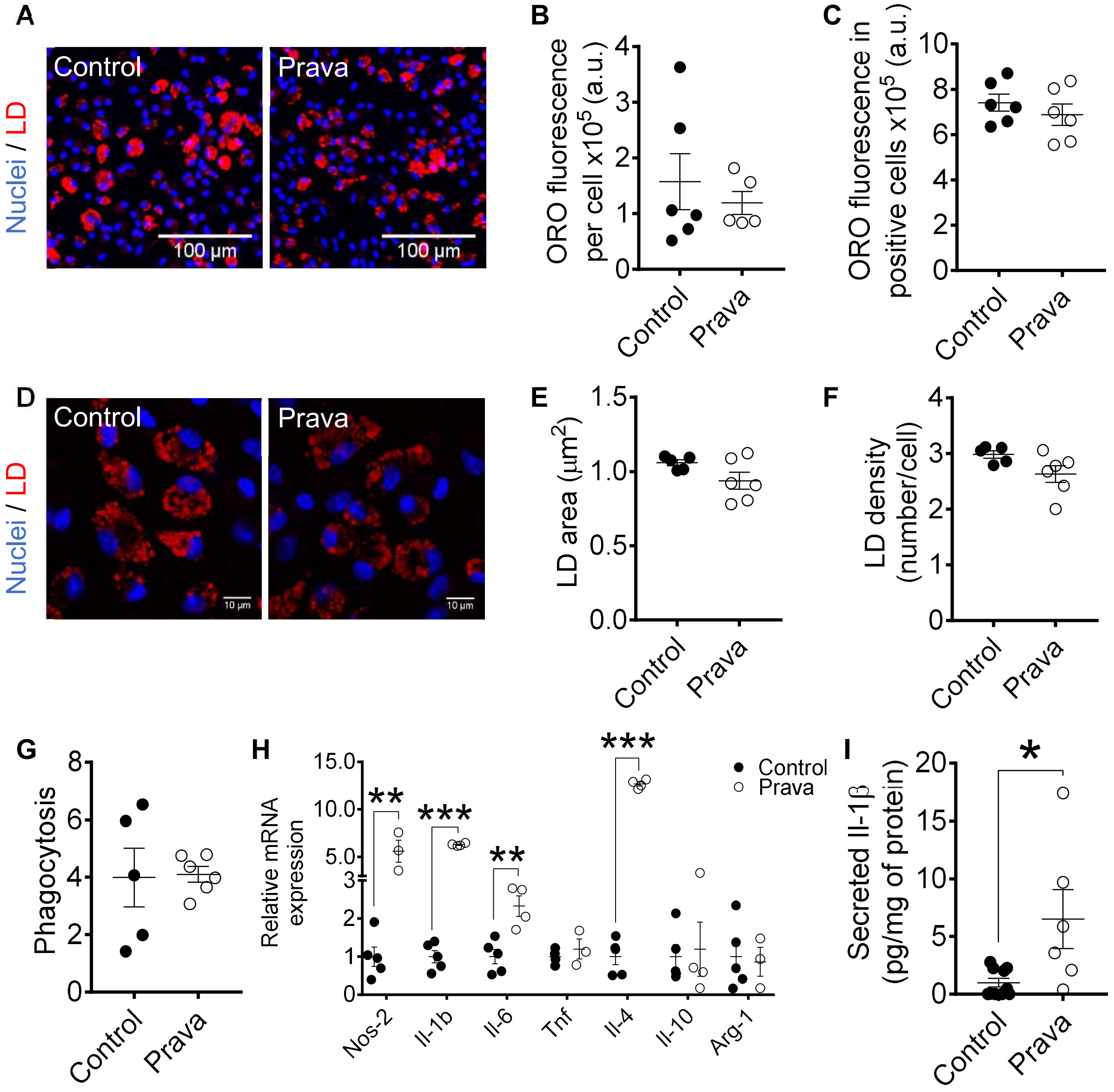

Supplementary Figure S10. Pravastatin treatment does not affect foam cell formation, lipid droplet density, phagocytosis and inflammatory gene expression in PM. (A) Representative images at 10x magnification of PM from non-treated (Control) and pravastatin-treated (Prava) LDLr-/- mice after incubation with ox-LDL. Neutral lipids were stained with ORO fluorescent dye (red) and nuclei with Hoechst33342 (blue). Quantitative analysis of ORO fluorescence intensity in all cells **(B)** and in positive cells **(C)**. Control (n=5 mice) and Prava (n=6 mice). **(D)** Representative images of lipid droplets at 60x magnification and stained with ORO (red) and nuclei with Hoechst33342 (blue). Quantitative analysis of lipid droplets' size **(E)** and density **(F)** from images displayed in the panel D. Three replicates per mouse, each corresponding to the average of nine fields analyzed by fluorescence microscopy. Control (n=5 mice) and Prava (n=6 mice). **(G)** Zymosan phagocytosis. Two replicates per mouse. Control (n=5 mice) and Prava (n=6 mice). **(H)** Relative mRNA expression of inflammatory related genes. Control (n=5 mice) and LDLr-/- (n=4 mice), with 2 replicates for each gene. Data are expressed as Mean ± SE.
(I) Interleukin-1β (Il-1β) secretion in cell supernatant and normalized by protein content in the cell lysate. Data are expressed as Mean ± SE. Control (n=10 mice) and Prava (n=6 mice), with 2 replicates for each mouse. Statistical analyses were performed using a two-tailed unpaired Student-t-test. *, **, *** with $p<0.05$, 0.01 and 0.001, respectively.

Supplementary Table S1. Oligonucleotides used to assess gene expression by RT-qPCR.

Gene	Transcript variant accession number*	Forward sequence (5'→3')	Reverse sequence (5'→3')	Amplicon size (bp)
Dnm1l	NM_001360007.1	ATGCCAGCAAGTCCACAGAA	TGTTCTCGGGCAGACAGTTT	86
Fis1	NM_025562.3	CAAAGAGGAACAGCGGGACT	ACAGCCCTCGCACATACTTT	95
Ppif	NM_134084.1	TGGCTCTCAGTTCTTTATCT	ACATCCATGCCCTCTTT	90
Mfn1	NM_024200.4	GCAGACAGCACATGGAGAGA	GATCCGATTCCGAGCTTCCG	83
Mfn2	NM_001285920.1	TGCACCGCCATATAGAGGAAG	TCTGCAGTGAACTGGCAATG	78
Opa1	NM_001199177.1	ACCTTGCCAGTTTAGCTCCC	TTGGGACCTGCAGTGAAGAA	82
Nos2	NM_010927.4	GTTCTCAGCCCAACAATACAAGA	GTGGACGGGTCGATGTCAC	127
Il-1b	NM_008361.4	CCTTCCAGGATGAGGACATGA	TGAGTCACAGAGGATGGGCTC	71
Il-6	NM_031168.2	CACGGCCTTCCCTACTTCAC	GGTCTGTTGGGAGTGGTATC	66
Tnf	NM_013693.3	CCCTCCTGGCCAACGGCATG	TCGGGGCAGCCTTGTCCCTT	109
Il-4	NM_021283.2	CCAAACGTCCTCACAGCAAC	AAGCCCGAAAGAGTCTCTGC	157
Il-10	NM_010548.2	GCTCTTACTGACTGGCATGAG	CGCAGCTCTAGGAGCATGTG	105
Arg-1	NM_007482.3	CTCCAAGCCAAAGTCCTTAGAG	AGGAGCTGTCATTAGGGACATC	185
Rplp0	NM_007475.5	GAGGAATCAGATGAGGATATGGGA	AAGCAGGCTGACTTGGTTGC	72
Actb	NM_007393.5	AGAAGCTGTGCTATGTTGCTCTA	TCAGGCAGCTCATAGCTCTTC	91

(*) Accession number of the *Mus musculus* longest transcript variant deposited in the nucleotide data bank of the National Center for Biotechnology and Information (NCBI), available online at https://www.ncbi.nlm.nih.gov/ and used as template to design oligonucleotides for RT-qPCR.
