# Supplementary material for: In Vivo Pravastatin Treatment Reverses Hypercholesterolemia Induced Mitochondria-Associated Membranes Contact Sites, Foam Cell Formation, and Phagocytosis in Macrophages
Source: Front Mol Biosci. 2022 Mar 15;9:839428. doi: 10.3389/fmolb.2022.839428 (PMC8965079; doi:10.3389/fmolb.2022.839428)
Supplement: Supplementary file 2 [file DataSheet2.docx]

**Assis et al. Frontiers Molec Biosci. 2022. Supplementary Material**

Nuclei / PLA

**B**

**D**


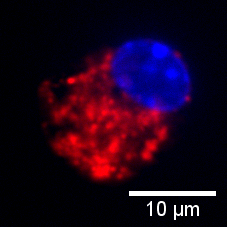

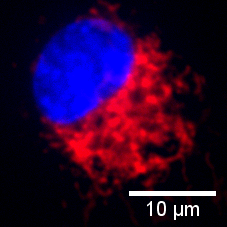


WT

LDLr-/-

Nucleus / Mitochondria

**C**

**E**

**F**


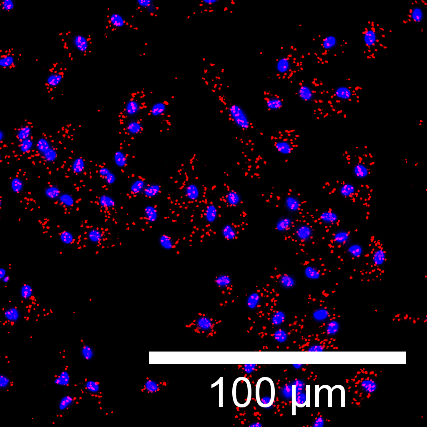

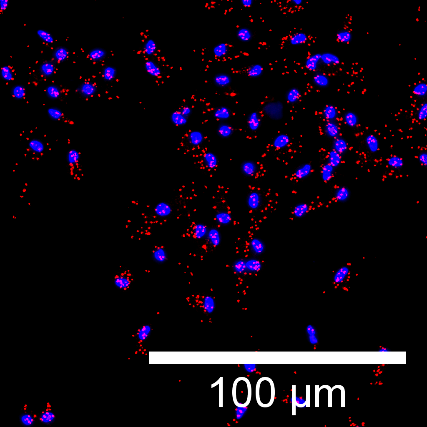


LDLr-/-

WT

**A**

**Supplementary Figure S1. Hypercholesterolemia increases mitochondrial branching in PM. (A)** Representative proximity ligation assay (PLA) images at 40x magnification and **(B)** quantitative analysis of Ip3r1-Vdac1 interactions in PM from WT and LDLr-/- mice. Ip3r1‑Vdac1 interactions detected through PLA assay were labeled with Cyanine 5 (red) and nuclei with DAPI (blue). Two replicates per mouse, each corresponding to the average of nine fields analyzed by fluorescence microscopy. WT (n=12 mice) and LDLr-/- (n=8 mice). **(C)** Representative images of mitochondria network at 60x magnification and quantitative analysis of mitochondria aspect ratio **(D)** and form factor **(E)**. Four replicates per mouse, each corresponding to the average of 9 fields analyzed by fluorescence microscopy. Mitochondria stained with MitoTracker (red) and nuclei with Hoechst 33342 (blue). WT (n=12 mice) and LDLr-/- (n=8 mice). **(F)** Relative gene expression of mitochondrial fusion and fission markers. WT (n=6 mice) and LDLr-/- (n=5 mice). Data are expressed as Mean ± SE. Statistical analyses were performed using a two-tailed unpaired Student-t test. * with p<0.05.

**A**

**B**

**C**

**D**

**E**

**F**

**G**

**H**

**I**

**Supplementary Figure S2. Hypercholesterolemia does not affect mitochondrial respiration and glycolytic function in BMDM. (A)** Average curves of oxygen consumption rates (OCR) of BMDM from WT and LDLr-/- mice. Oligomycin (O), FCCP, rotenone plus antimycin-A (R+A) were sequentially injected to assess mitochondrial respiratory rates associated to specific states: **(B)** basal respiration, **(C)** ATP production, **(D)** maximal respiration, **(E)** proton leak and **(F)** non‑mitochondrial oxygen consumption. **(G)** Average curves of extracellular acidification rate (ECAR) of BMDM from WT and LDLr-/- mice. **(H)** Glycolysis and **(I)** glycolytic capacity. OCR and ECAR values were normalized by the respective DNA amount in each well. Three replicates per mouse. Data are expressed as Mean ± SE. WT (n=5 mice) and LDLr-/- (n=6 mice) for OCR and ECAR assays. Statistical analyses were performed using a two‑tailed unpaired Student-t test.

**A**

**B**

**C**

**D**

**E**

**F**

**G**

**H**

**I**

**Supplementary Figure S3. Hypercholesterolemia does not affect mitochondrial respiration and glycolytic function in PM. (A)** Average curves of oxygen consumption rates (OCR) of PM from WT and LDLr-/- mice. Oligomycin (O), FCCP, rotenone plus antimycin-A (R+A) were sequentially injected to assess mitochondrial respiratory rates associated to specific states: **(B)** basal respiration, **(C)** ATP production, **(D)** maximal respiration, **(E)** proton leak and **(F)** non‑mitochondrial oxygen consumption. WT (n=6 mice) and LDLr-/- (n=4 mice). **(G)** Average curves of extracellular acidification rate (ECAR) of PM from WT and LDLr-/- mice. **(H)** Glycolysis and **(I)** glycolytic capacity. WT (n=6 mice) and LDLr-/- (n=3 mice). OCR and ECAR values were normalized by the respective DNA amount in each well. Three replicates per mouse. Data are expressed as Mean ± SE. Statistical analyses were performed using a two‑tailed unpaired Student-t test.

**A**

**B**

**C**

**D**

**E**

**F**

**G**

**H**

**I**

**Supplementary Figure S4. Hypercholesterolemia reduces global superoxide anion production and modulates hydrogen peroxide release in PM.** Detection of global **(A)** and mitochondria-derived **(B)** superoxide anion production in PM from WT and LDLr-/- mice. Four replicates per mouse, each corresponding to the average of 9 fields analyzed by fluorescence microscopy. WT (n=12 mice) and LDLr‑/- (n=8 mice). Average curves and rate quantitation of total **(C,D)**, non‑mitochondrial **(E,F)** and mitochondrial **(G,H)** release of hydrogen peroxide (H_2_O_2_). Values were normalized by DNA amount in each well. Three replicates per mouse. Data are expressed as Mean ± SE. WT (n=8 mice) and LDLr‑/- (n=7 mice). **(I)** Oxidative stress assessed by GSH/GSSG ratio. WT (n=5 mice) and LDLr-/- (n= 5 mice). Statistical analyses were performed using a two‑tailed unpaired Student-t test. *, **, *** with p<0.05, 0.01 and 0.01, respectively.

**A**

**B**

**C**

**D**

**E**

**F**

**G**

**H**


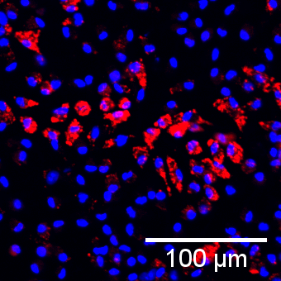

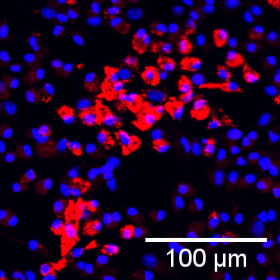


WT

LDLr-/-

Nuclei / LD


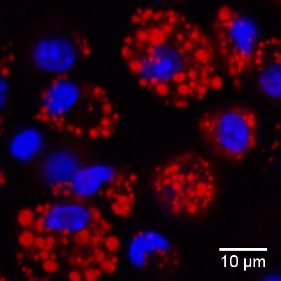

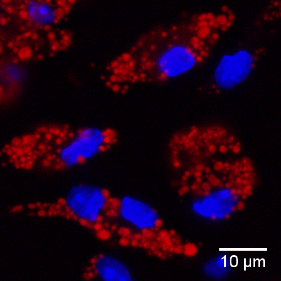


Nuclei / LD

WT

LDLr-/-

**Supplementary Figure S5. Hypercholesterolemia modulates pro- and anti-inflammatory gene expression in PM.** **(A)** Representative images at 10x magnification of PM from WT and LDLr‑/- mice after incubation with ox-LDL. Neutral lipids were stained with ORO fluorescent dye (red) and nuclei with Hoechst33342 (blue). Quantitative analysis of ORO fluorescence intensity in all cells **(B)** and in LD positive cells **(C)**. **(D)** Representative images of lipid droplets at 60x magnification and stained with ORO (red) and nuclei with Hoechst33342 (blue). Quantitative analysis of lipid droplets’ size **(E)** and density **(F)** from images displayed in the panel D. Three replicates per mouse, each corresponding to the average of nine fields analyzed by fluorescence microscopy. WT (n=6 mice) and LDLr-/- (n=5 mice). **(G)** Zymosan phagocytosis. WT (n=5 mice) and LDLr-/- (n=4 mice). Two replicates per mouse. **(H)** Relative mRNA expression of inflammatory related genes. WT (n=6 mice) and LDLr-/- (n=5 mice), with 2 replicates for each gene. Data are expressed as Mean ± SE. Statistical analyses were performed using two‑tailed unpaired Student-t-test. *, ** with p<0.05 and 0.01, respectively.

Nuclei / PLA

Nuclei / Mitochondria

**B**

**D**

**E**

**F**

**G**

**H**


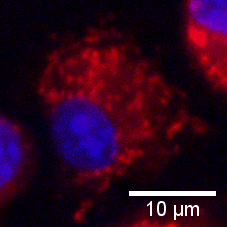

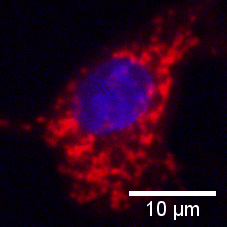


Control

Prava

**C**


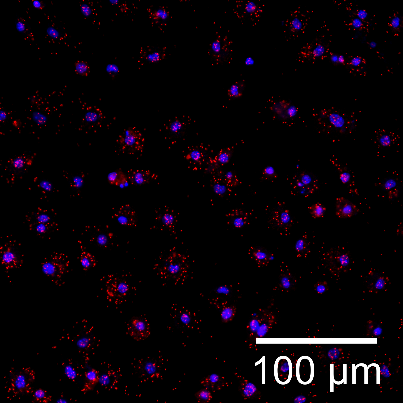

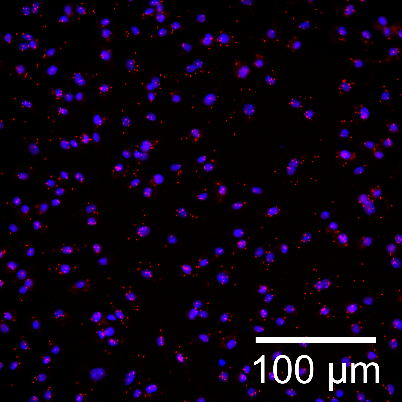


Prava

Control

**A**

**Supplementary Figure S6. Pravastatin treatment decreases ER-mitochondria interaction and upregulates Fis1 and Mfn2 gene expression in PM.** **(A)** Representative proximity ligation assay (PLA) images at 40x magnification and **(B)** quantitative analysis of Ip3r1-Vdac1 interactions in PM from non-treated (Control) and pravastatin-treated (Prava) LDLr-/- mice. Ip3r1-Vdac1 interactions detected through PLA assay were labeled with Cyanine 5 (red) and nuclei with DAPI (blue). Two replicates per mouse, each corresponding to the average of nine fields analyzed by fluorescence microscopy. Control (n=6 mice) and Prava (n=4 mice). **(C)** Representative images of mitochondria network at 60x magnification and quantitative analysis of mitochondria aspect ratio **(D)** and form factor **(E)**. Four replicates per mouse, each corresponding to the average of 9 fields analyzed by fluorescence microscopy. Mitochondria stained with MitoTracker (red) and nuclei with Hoechst 33342 (blue). Control (n=8 mice) and Prava (n=6 mice). Statistical analyses were performed using a two-tailed unpaired Student-t test. *p<0.05. Correlation analyses between Ip3r1-Vdac1 interactions detected by PLA and mitochondria aspect ratio **(F)** and form factor **(G)**. Control (open circles) and Prava (filled circles). Statistical analyses using Person’s correlation test. **(H)** Relative gene expression of mitochondrial fusion and fission markers. Control (n=5 mice) and Prava (n=4 mice). Data are expressed as Mean ± SE. Statistical analyses were performed using a two-tailed unpaired Student-t test. *, ***with p<0.05 and 0.001, respectively.

**A**

**B**

**C**

**D**

**E**

**F**

**G**

**H**

**I**

**Supplementary Figure S7. Pravastatin treatment does not affect mitochondrial respiration and glycolytic function in BMDM. (A)** Average curves of oxygen consumption rates (OCR) of BMDM from non-treated (Control) and pravastatin-treated (Prava) LDLr-/- mice. Oligomycin (O), FCCP, rotenone plus antimycin-A (R+A) were sequentially injected to assess mitochondrial respiratory rates associated to specific states: **(B)** basal respiration, **(C)** ATP production, **(D)** maximal respiration, **(E)** proton leak and **(F)** non-mitochondrial oxygen consumption. **(G)** Average curves of extracellular acidification rate (ECAR) of BMDM from Control and Prava mice. **(H)** Glycolysis and **(I)** glycolytic capacity. OCR and ECAR values were normalized by the respective DNA amount in each well. Three replicates per mouse. Data are expressed as Mean ± SE. Control (n=4 mice) and Prava (n=4 mice) for OCR and ECAR assays. Statistical analyses were performed using a two‑tailed unpaired Student-t test.

**A**

**B**

**C**

**D**

**E**

**F**

**G**

**H**

**I**

**Supplementary Figure S8. Pravastatin treatment reduces mitochondrial respiration in PM. (A)** Average curves of oxygen consumption rates (OCR) of PM from non-treated (Control) and pravastatin-treated (Prava) LDLr-/- mice. Oligomycin (O), FCCP, rotenone plus antimycin-A (R+A) were sequentially injected to assess mitochondrial respiratory rates associated to specific states: **(B)** basal respiration, **(C)** ATP production, **(D)** maximal respiration, **(E)** proton leak and **(F)** non-mitochondrial oxygen consumption. **(G)** Average curves of extracellular acidification rate (ECAR) of PM from Control and Prava mice. **(H)** Glycolysis and **(I)** glycolytic capacity. OCR and ECAR values were normalized by the respective DNA amount in each well. Three replicates per mouse. Data are expressed as Mean ± SE. Control (n=5 mice) and Prava (n=5 mice) for OCR and ECAR assays. Statistical analyses were performed using a two‑tailed unpaired Student-t test. * with p<0.05.

**A**

**B**

**C**

**D**

**E**

**F**

**G**

**H**

**I**

**Supplementary Figure S9. Pravastatin treatment increases global superoxide anion production in PM.** Detection of global **(A)** and mitochondria-derived **(B)** superoxide anion production in PM from non-treated (Control) and pravastatin-treated (Prava) LDLr-/- mice. Four replicates per mouse, each corresponding to the average of 9 fields analyzed by fluorescence microscopy. Control (n=5 mice) and Prava (n=5 mice). Average curves and rate quantitation of total **(C,D)**, non‑mitochondrial **(E,F)** and mitochondrial **(G,H)** release of hydrogen peroxide (H_2_O_2_). Values were normalized by DNA amount in each well. Three replicates per mouse. Data are expressed as Mean ± SE. Control (n=11 mice) and Prava (n=8 mice). **(I)** Oxidative stress assessed by GSH/GSSG ratio. Control (n=5 mice) and Prava (n=4 mice). Statistical analyses were performed using a two‑tailed unpaired Student-t test. * with p<0.05 respectively.

**A**

**B**

**C**

**D**

**E**

**F**

**G**

**H**

**I**


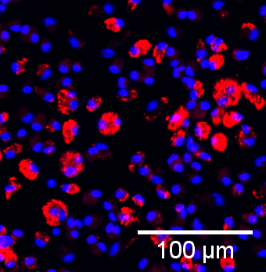

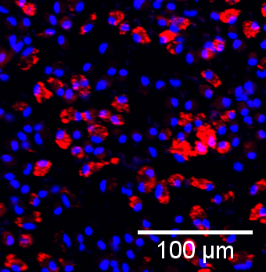


Control

Prava

Nuclei / LD


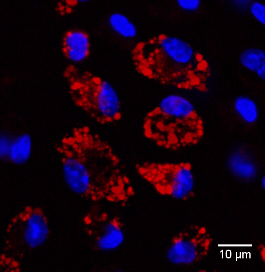

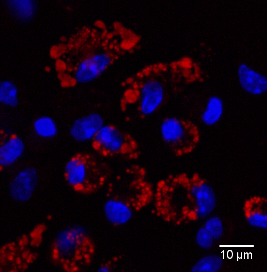


Control

Prava

Nuclei / LD

**Supplementary Figure S10. Pravastatin treatment does not affect foam cell formation, lipid droplet density, phagocytosis and inflammatory gene expression in PM. (A)** Representative images at 10x magnification of PM from non-treated (Control) and pravastatin-treated (Prava) LDLr‑/- mice after incubation with ox-LDL. Neutral lipids were stained with ORO fluorescent dye (red) and nuclei with Hoechst33342 (blue). Quantitative analysis of ORO fluorescence intensity in all cells **(B)** and in positive cells **(C)**. Control (n=5 mice) and Prava (n=6 mice). **(D)** Representative images of lipid droplets at 60x magnification and stained with ORO (red) and nuclei with Hoechst33342 (blue). Quantitative analysis of lipid droplets’ size **(E)** and density **(F)** from images displayed in the panel D. Three replicates per mouse, each corresponding to the average of nine fields analyzed by fluorescence microscopy. Control (n=5 mice) and Prava (n=6 mice). **(G)** Zymosan phagocytosis. Two replicates per mouse. Control (n=5 mice) and Prava (n=6 mice). **(H)** Relative mRNA expression of inflammatory related genes. Control (n=5 mice) and LDLr-/- (n=4 mice), with 2 replicates for each gene. Data are expressed as Mean ± SE. **(I)** Interleukin-1β (Il-1β) secretion in cell supernatant and normalized by protein content in the cell lysate. Data are expressed as Mean ± SE. Control (n=10 mice) and Prava (n=6 mice), with 2 replicates for each mouse. Statistical analyses were performed using a two‑tailed unpaired Student-t-test. *, **, *** with p<0.05, 0.01 and 0.001, respectively.

**Supplementary Table S1. Oligonucleotides used to assess gene expression by RT‑qPCR.**

| **Gene** | **Transcript variant accession number*** | **Forward sequence (5'→3')** | **Reverse sequence (5'→3')** | **Amplicon size (bp)** |
| --- | --- | --- | --- | --- |
| Dnm1l | NM_001360007.1 | ATGCCAGCAAGTCCACAGAA | TGTTCTCGGGCAGACAGTTT | 86 |
| Fis1 | NM_025562.3 | CAAAGAGGAACAGCGGGACT | ACAGCCCTCGCACATACTTT | 95 |
| Ppif | NM_134084.1 | TGGCTCTCAGTTCTTTATCT | ACATCCATGCCCTCTTT | 90 |
| Mfn1 | NM_024200.4 | GCAGACAGCACATGGAGAGA | GATCCGATTCCGAGCTTCCG | 83 |
| Mfn2 | NM_001285920.1 | TGCACCGCCATATAGAGGAAG | TCTGCAGTGAACTGGCAATG | 78 |
| Opa1 | NM_001199177.1 | ACCTTGCCAGTTTAGCTCCC | TTGGGACCTGCAGTGAAGAA | 82 |
| Nos2 | NM_010927.4 | GTTCTCAGCCCAACAATACAAGA | GTGGACGGGTCGATGTCAC | 127 |
| Il-1b | NM_008361.4 | CCTTCCAGGATGAGGACATGA | TGAGTCACAGAGGATGGGCTC | 71 |
| Il-6 | NM_031168.2 | CACGGCCTTCCCTACTTCAC | GGTCTGTTGGGAGTGGTATC | 66 |
| Tnf | NM_013693.3 | CCCTCCTGGCCAACGGCATG | TCGGGGCAGCCTTGTCCCTT | 109 |
| Il-4 | NM_021283.2 | CCAAACGTCCTCACAGCAAC | AAGCCCGAAAGAGTCTCTGC | 157 |
| Il-10 | NM_010548.2 | GCTCTTACTGACTGGCATGAG | CGCAGCTCTAGGAGCATGTG | 105 |
| Arg-1 | NM_007482.3 | CTCCAAGCCAAAGTCCTTAGAG | AGGAGCTGTCATTAGGGACATC | 185 |
| Rplp0 | NM_007475.5 | GAGGAATCAGATGAGGATATGGGA | AAGCAGGCTGACTTGGTTGC | 72 |
| Actb | NM_007393.5 | AGAAGCTGTGCTATGTTGCTCTA | TCAGGCAGCTCATAGCTCTTC | 91 |

(*) Accession number of the *Mus musculus* longest transcript variant deposited in the nucleotide data bank of the National Center for Biotechnology and Information (NCBI), available online at https://www.ncbi.nlm.nih.gov/ and used as template to design oligonucleotides for RT-qPCR.
